# Burden of catastrophic costs, income inequalities and associated factors in Guatemala: First national tuberculosis costs survey, 2023

**DOI:** 10.1371/journal.pone.0351164

**Published:** 2026-08-17

**Authors:** Hibeb Alejandra Silvestre Tuch, Maritza Samayoa-Peláez, Belkys Asuncion Marcelino Martinez, Ernesto Montoro, Pedro Avedillo, Barbara Reis-Santos

**Affiliations:** 1 Tuberculosis Program, Ministry of Health and Social Assistance of Guatemala, Guatemala City, Guatemala; 2 Pan American Health Organization, Washington D. C., United States of America; 3 Autonomous researcher, São Paulo, São Paulo, Brazil; Liverpool School of Tropical Medicine, UNITED KINGDOM OF GREAT BRITAIN AND NORTHERN IRELAND

## Abstract

**Objectives:**

To estimate the prevalence of catastrophic costs incurred by households of people with tuberculosis (TB) in Guatemala, and to assess the potential wealth inequalities and the associated factors with the catastrophic costs, social consequences and coping strategies.

**Methods:**

A national representative survey, employing a clustered sampling based on health facilities, was conducted in 2023. The participants were people with TB and the data was collected by trained professionals using a standardized questionnaire. Catastrophic costs were defined as costs incurred by TB treatment that exceeds 20% of annual household income. We estimated the burden of catastrophic costs, social consequences and coping strategies. The primary analysis encompassed the output approach, while sensitivity analyses were conducted with the human capital approach and income loss adjustments. The associations were assessed using Poisson regression models with robust variance.

**Results:**

The First Guatemala National Tuberculosis Costs Survey had 530 participants enrolled in 42 clusters. The estimated catastrophic costs incurred by people with TB, through the output approach, was 36% (95% CI 29% − 43%). The prevalence among the poorest wealth quintile was 66%, while among the richest it was 50%, throughout the output approach adjusted for income loss (sensitivity analysis). Catastrophic costs were twice as high for 15–44-years old, but lower for people in the South West (PR 0.72; 95% CI 0.55–0.94), those in five-plus-person households (PR 0.73; 95% CI 0.57–0.93), and those treated in primary/secondary facilities (PR 0.69; 95% CI 0.49–0.97). Social effects were related with sociodemographic characteristics and catastrophic costs (PR 1.21; 95% CI 1.09–1.34). Coping strategies were associated with sociodemographic factors, presence of social support (PR 1.11; 95% CI 1.01–1.22), and TB type (PR 1.16; 95% CI 1.01–1.33).

**Conclusion:**

In Guatemala, catastrophic costs were identified as a burden characterized by inequities, posing a challenge to the country in achieving zero to this indicator. However, the analyses revealed pathways that could be prioritized when formulating policies to effectively address this issue.

## Introduction

It is widely acknowledged that no person should experience catastrophic – that is, costs which exceeds 20% of their annual household income – related to tuberculosis (TB) treatment. Notably, this recognition has been incorporated into the impact targets of the End TB Strategy of the World Health Organization (WHO) [1].

From 2015 to 2024, 37 countries conducted national surveys to estimate the costs incurred by people with TB and their households, and 35 of these reported their results to the WHO. The pooled average for all 35 countries, weighted according to the number of notified cases for each country, was 49% [95% confidence interval (CI) 38% − 60%], ranging from 13% (95% CI 10% − 17%) in El Salvador to 92% (95% CI 86% − 97%) in the Solomon Islands [2].

In the Americas region, the number of countries conducting national surveys to estimate catastrophic costs that have been validated by the WHO is notably low [2]. It is noteworthy that, in Central America, only El Salvador (data presented above) has published data on these initiatives. The reported results from Argentina, Brazil and Colombia estimated percentages of catastrophic costs of 48% (95% CI 41% − 56%), 48% (95% CI 43% − 53%) and 52% (95% CI 45% − 58%), respectively [2–4]. Achieving the WHO goal of zero catastrophic costs, both in the region and within countries, may be delayed by limited knowledge of the burden of this indicator. However, it is important to highlight that the use of data from other countries, published estimates, and out-of-pocket expenditure data are strategies that can contribute to the formulation and adoption of policies to mitigate the burden of catastrophic costs, accelerating the achievement of the goal [2,5,6].

In 2023, Guatemala presented an estimated TB incidence of 33 per 100,000 population, corresponding to 5,900 new TB cases, and a TB case fatality ratio of 10% [2]. Nevertheless, with respect to the End TB Strategy milestones of a 50% reduction in the TB incidence rate and a 75% reduction in the total number of TB deaths compared with 2015 by 2025, the country demonstrated a 27% increase for both. Furthermore, no published data on catastrophic costs has been made available. Given this gap, and the need for reliable evidence to inform policy and social protection efforts, Guatemala undertook its first national TB costs survey in 2023.

The objective of this study was to estimate the prevalence of catastrophic costs incurred by households of people with TB in Guatemala, and to assess the potential wealth inequalities and the associated factors with the catastrophic costs, social consequences and coping strategies.

## Methods

### Design

A cross-sectional study was conducted to assess the burden of catastrophic costs, wealth inequalities and associated factors among people with TB in Guatemala, between August 14 to September 8, 2023. The survey was of a national representative nature, employing a clustered sampling method and based on health facilities. The design and methods were developed in accordance with the Handbook on National TB Patient Cost Surveys of the WHO [7], and adapted to Guatemala.

### Setting

Guatemala is a Central American country with an area of 108,888 km² and a population of 18 million. The geographical situation of the country is defined by its borders with Mexico to the north and west, Belize to the northeast, Honduras to the east, and El Salvador to the southeast. Guatemala is the largest economy in Central America and is classified as an upper middle-income country, as indicated by its GDP per capita (US $4,603 in 2020) [8]. The Health System of Guatemala is comprised of both public and private sectors. However, despite government participation in the health sector, the out-of-pocket expenditure as a percentage of current health expenditure reached 58.4 in Guatemala, in 2022, the highest in the Americas and more than twice the estimated average for the region (23.3%) [6]. The Ministry of Health and Social Assistance of Guatemala and the Guatemalan Institute of Social Security are the two entities with the responsibility of overseeing the public sector, which provide people with TB with treatment at no cost to the individual.

### Participants

The study participants were composed of people with a diagnosis of TB, either susceptible or drug-resistant (DR-TB), who were under treatment in a health facility of the Ministry of Health and Social Assistance of Guatemala and with a minimum of 14 days of treatment, either in the intensive or continuation phase, irrespective of age. The exclusion criteria encompassed individuals under the age of 18 without a designated tutor or companion, those experiencing homelessness, and those subjected to circumstances of deprivation of liberty. The latter two are due to operational considerations regarding outreach to the public and ensuring adequate representation.

### Data collection and measurements

The enrolment of eligible participants at the selected health facilities was conducted from August 14 to September 8, 2023. The data was collected by trained health professionals using a standardized electronic questionnaire, under the guidance of a consultant team, the Tuberculosis Program and Pan American Health Organization. This questionnaire was developed by the WHO and adapted to Guatemala, and the ONA web-based data capture platform (Ona Systems Inc.) was used as the data collection tool.

The questionnaire was divided into four sections, providing information on sociodemographic and clinical characteristics, employment status, household composition, individual and household income, healthcare utilization, TB treatment-related costs, time spent and income lost while seeking and receiving care, caregiver time, health and social protection measures, coping mechanisms, and social consequences [7].

The detailed methods of the assessment of the variables are described in the World Health Organization’s Handbook on National TB Patient Cost Surveys [7].

**Fig 1:**
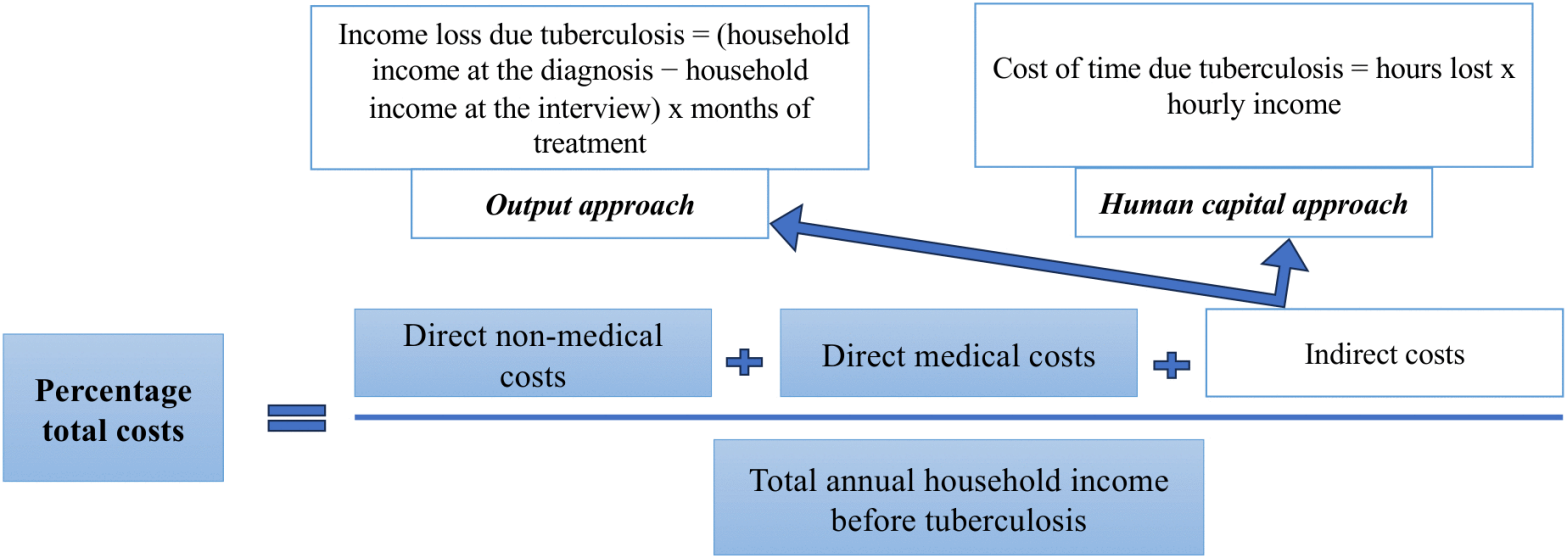
Determining the percentage total costs incurred by people with tuberculosis and their households, in relation to their household income; First Guatemala national tuberculosis costs survey, 2023. Adapted from Consolidated guidance on tuberculosis data generation and use [9].

Direct costs were defined as direct payments made by survey participants while seeking or accessing TB care. This encompasses both medical costs, including the costs of bed charges, consultation fees, imaging tests, medicines, laboratory tests, or other medical procedures, and non-medical costs, such as transportation, food while seeking medical care, nutritional supplements, accommodation, among other expenses not directly clinical related [5]. With regard to the indirect costs, the output approach (an estimate of income loss) involved the use of self-reported household income at the time of diagnosis and during the current treatment phase to estimate the difference in income before and during the current TB episode (Fig 1) [7]. The human capital approach (an estimate of the cost of time lost) entailed the use of self-reported time (in hours) spent either seeking or receiving TB care, multiplied by an individual hourly income, to estimate the cost of time lost (Fig 1) [7].

The study participants reported individual and household income prior to the diagnosis of TB and at the time of interview. The total annual household income was estimated by both self-reported household income and estimated household annual income based on household assets and dwelling characteristics [7,9]. For the estimation of income based on assets, the variables from the National Survey of Employment and Income (ENEI) in 2021, as conducted by the National Institute of Statistics (INE) of Guatemala, were selected for inclusion in the survey through a multivariate linear regression model.

The poverty line was defined as an income of less than US$1.90 per day in terms of purchasing power parity [8]. The monetary values were expressed in Guatemalan quetzales and subsequently converted to US dollars at an exchange rate of US$1 = 7.63, which was the average rate for the year 2023 [8].

When the TB diagnosis was performed from four weeks after the onset of symptoms, it was categorized as a TB diagnosis delay.

### Variables

The percentage of total costs incurred by individuals treated for TB and their households that exceeds 20% of their annual household income (catastrophic costs main: no; yes) [7,9], an End TB Strategy indicator, was the primary dependent study variable. The percentage of total costs was calculated by dividing the sum of direct medical, direct non-medical, and income loss (indirect costs) divided by the total annual household income before tuberculosis, according to the most recent WHO recommendations and data availability Fig 1 [9]. The indirect costs were estimated through the output and human capital approaches for the main and sensitivity analyses, respectively [9].

Social effects refer to self-reporting of at least one of the following: food insecurity, job losses, social exclusion, divorce or children dropping out of school (any social effects: no; yes). Coping strategies encompassed the asset sale, borrowing, and/or other measures (coping strategies: no; yes). In the association analyses, both variables were evaluated as secondary outcomes, employing a binary approach.

In order to characterize the population and to explore the factors associated with the catastrophic costs, any social effect and coping strategies, we included sociodemographic variables: sex (female; male), age group (0–14; 15–44; 45–64; 65+), educational level (no education; primary school; secondary school or higher), employed before TB and at the time of the interview (no; yes), sector of labor market (formal; informal), below poverty line (no; yes), social support [cases where participants or their families receive social assistance following a TB diagnosis, including sick leave, disability benefits, cash transfers, or other forms of assistance (no; yes)], health insurance (no; yes), region of residence (Metropolitan – I; North – II; North East – III; South East – IV; Central – V; South West – VI; North West – VII; Peten – VIII), and household size (0–4; 5+). The clinical and health related variables were: HIV positivity (no; yes), other comorbidities (no; yes), previous TB (no; yes), DR-TB (no; yes), type of TB (bacteriologically confirmed pulmonary; clinically diagnosed pulmonary; extrapulmonary), TB diagnosis delay (no; yes), ever hospitalized during current TB treatment (no; yes), health facility type (tertiary care; primary/secondary care), treatment phase (intensive; continuation), and self-administrated treatment (no; yes).

### Bias handling

A series of measures were undertaken in order to reduce the presence of information bias in the survey. These measures included the administration of a standardized and validated questionnaire; the implementation of theoretical and practical training for all interviewers; the conduction of a pilot study to verify the functionality of the data collection instrument, the interview time and procedures, and the technical and logistical difficulties; and the implementation of quality control of data entry.

The pilot study comprised 19 people with TB from two healthcare facilities, and the results were not incorporated into the study. The findings of this evaluation underscored the necessity for modifications to both the application and the operational process for collecting information.

In quality control, a daily supervision plan was implemented to ensured accurate data entry. An analysis and cleaning process was also performed. Subsequently, the data was verified using analytical methods, employing conditional formats, validation grids, and cross-tabulations to ensure their consistency and reliability.

Furthermore, the participants during the intensive phase of TB treatment (initial phase) reported information about the current and the pre-treatment phase, while those at the continuation phase reported data only about the current phase. This strategy was employed in an attempt to minimize the recall bias.

### Study size and sampling

A sample size calculation was conducted based on 3,151 reported cases of TB in Guatemala between 3 May 2021 and 25 May 2022. The study employed a design effect of 1.5 – accounting for an intra-cluster correlation of 4% and a cluster size of 13 participants, a precision level of 5%, a participation rate of 90%, and a proportion of households facing catastrophic costs of 50%. The resulted sample sizes of 571 participants encompassed 44 clusters. The health facilities were the primary sampling units, and were sampled randomly with probability proportional to size, accounting to regional strata. Consequently, the sampling process reflected the proportional allocation of the cases of TB across the eight geographic regions of Guatemala. The enrolment of participants was conducted in a consecutive basis with people with TB who were attending follow-up visits, until the stipulated number had been reached.

### Statistical methods

The analytical process was aligned with the recommendations and standardized procedures outlined by the WHO. The results reported were obtained by employing the R 4.4.1 statistical software (Comprehensive R Archive Network). They were weighted according to the design effect and cluster sampling, by using sample expansion fact that considered the distribution and characteristics of the target population and study losses.

The costs for the remaining of the current treatment phase were extrapolated based on the total planned phase duration and number of phase days completed [7]. Consequently, the total cost of the phase was the product of the phase duration and the cost that has been incurred to date, divided by the time that has elapsed to date. With regard to the costs incurred by participants beyond the current treatment phase, these were based on the median costs incurred by other participants and their households in the alternative treatment phase at the time of the interview, in view of DR-TB status [7]. Inconsistencies and missing data in the income variables were addressed as outlined in Table 1, adapted from WHO recommendations [9], according to data availability.

**Table 1:** Description of the strategies implemented in order to address inconsistencies^a^ and missing data in income variables; First Guatemala national tuberculosis costs survey, 2023.

Estimate	Analysis	Employment status	Strategy implemented
**Output approach: indirect costs (income loss – numerator)**	Main	Employed before onset of TB	Missing data and inconsistencies on household income before or during TB treatment were handled by using the average household income for each time period. This was estimated based on data from other survey participants
		Not employed before onset of TB	Missing data and inconsistencies on household income before onset of TB or during TB treatment were replaced with zero
	Sensitivity 1	Current employed	Missing data and inconsistencies on household income during TB treatment were handled by using the average household income for the time period. This was estimated based on data from other survey participants. Missing data and inconsistencies on household income prior to the onset of TB were handled according to employment status at the time (main)
		Current not employed	Missing data and inconsistencies on household income during TB treatment were replaced with zero. Missing data and inconsistencies on household income prior to the onset of TB were handled according to employment status at the time (main)
**Human capital approach: indirect costs (time lost – numerator)**	Sensitivity 2	Employed before onset of TB	Missing data and inconsistencies on hourly income of an adult participant, or of the caregiver of a child, were handled by using the average wages based on data from other survey participants, according to labor sector (formal or informal)
		Not employed before onset of TB	Missing data and inconsistencies on hourly income of an adult participant, or of the caregiver of a child, were handled by using the hourly national minimum wages
	Sensitivity 3	Current employed	Missing data and inconsistencies on hourly income of an adult participant, or of the caregiver of a child, were handled by using the average wages based on data from other survey participants, according to labor sector (formal or informal)
		Current not employed	Missing data and inconsistencies on hourly income of an adult participant, or of the caregiver of a child, were handled by using the hourly national minimum wages
**Total annual household** **income before TB (denominator)**		Not applicable	Missing data and inconsistencies were handled by using regression analysis based on household asset ownership and dwelling characteristics

Adapted from Consolidated guidance on tuberculosis data generation and use [9].

a) Zeros were addressed as inconsistencies or not, depending on the employment situation and the estimate approach. In the output approach, zeros with unemployed status were retained and those with employed status were treated as inconsistencies. In the human capital approach, zeros were treated as inconsistencies.

TB: tuberculosis.

The estimation of the burden of catastrophic costs, along with the 95% CI, was determined by means of the output approach [9]. In order to conduct sensitivity analyses, the human capital approach was employed, and alternative catastrophic costs variables were built through the current employment status of the participants (Table 1) [9]. Consequently, when interpreted in a complementary manner, both the main analysis and the sensitivity analysis provide a more comprehensive understanding of the burden of TB catastrophic costs in Guatemala. The calculations were performed in accordance with the equations presented in Fig 1.

The descriptive analysis also included the distribution of frequencies, means and 95% CI, to the overall population and according to catastrophic costs status estimated by the output approach, for the covariates, in order to characterize the population. In addition, *equiplots* were constructed to illustrate the absolute inequality in catastrophic costs, burden, social effects, coping strategies and social support across household income quintiles before the onset of TB [10].

The association of catastrophic costs (output approach – the primary outcome) with sociodemographic, clinical and other health-related characteristics was assessed using Poisson regression models with robust variance [11]. These models were based on a previously published conceptual model [12] for which hierarchical levels were defined, from the most distal to the most proximal to the outcome. The model was composed of sets of variables, including sociodemographic, presence of comorbidities and history of TB, characteristics of current TB treatment, and social support for treatment. Thus, the independent variables were included in the regression models, according to the following hierarchical levels: level 1 (sex, age group, educational level, labor market sector, under poverty line, region of residence, household size, and health insurance), level 2 (HIV positivity, other comorbidities, and previous TB), level 3 (DR-TB, TB type, diagnosis delay, ever hospitalized, facility type, and self-administrated treatment), and level 4 (social support). Consequently, the effect of each characteristic on the catastrophic costs is interpreted as having been adjusted for all the variables that belong to the hierarchical levels below it, as well as for those on the same level [11]. This strategy was designed to minimize overadjustment. Despite the cross-sectional nature of the study, it can be posited that the independent variables incorporated within the model precede catastrophic costs, which are an outcome of treatment [12]. Furthermore, the hierarchical entry of independent variables into the model prevented adjustment by intermediate variables [12]. Given that the costs were extrapolated based on the treatment phase, each of the hierarchical levels was adjusted for this variable.

Moreover, the same model was employed to evaluate the association of the covariates with any social consequence and coping strategies. The hierarchical levels: level 1 (sex, age group, educational level, labor market sector, under poverty line, region of residence, household size, and health insurance), level 2 (HIV positivity, other comorbidities, and previous TB), level 3 (DR-TB, TB type, diagnosis delay, ever hospitalized, facility type, and self-administrated treatment), and level 4 (social support and catastrophic costs). The estimates were presented as prevalence ratios (PR) and 95% CI.

### Ethical approval

The protocol of the study was approved by the National Committee on Health Ethics of Guatemala under the opinion number CNES/017–2023, on May 31, 2023, and the Ethics Review Board of the Pan American Health Organization under the opinion number PAHOERC0665.03, on July 10, 2023. Written informed consent was obtained from all adults and legal guardians of minors during the interview.

## Results

**Fig 2:**
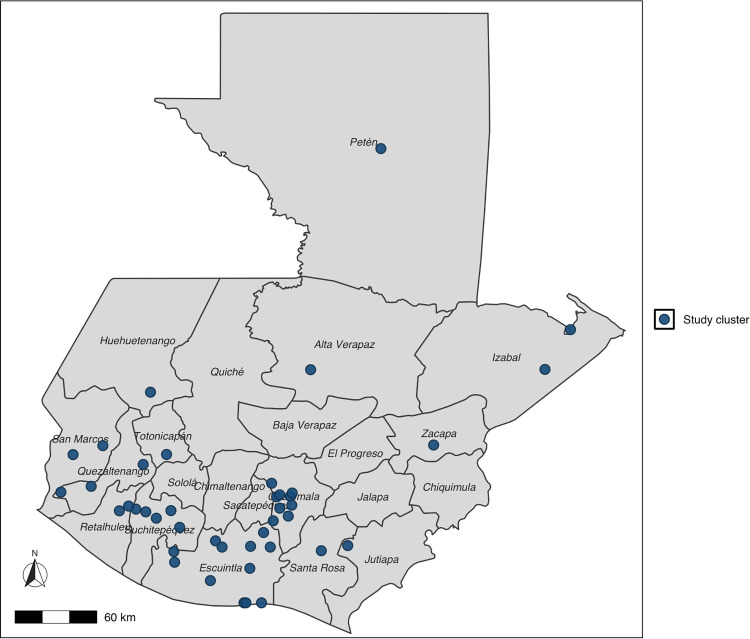
Spatial distribution of health facilities treating people with tuberculosis included in the study; First Guatemala national tuberculosis costs survey, 2023.

The First Guatemala National Tuberculosis Costs Survey, conducted in from August 14 to September 8, 2023, achieved a 93% response rate, with 530 participants enrolled in 42 clusters (Fig 2). The loss of clusters was due to two factors: first, the absence of persons with TB at the selected site at the time of data collection; and second, the absence of other centers in the same stratum that could replace it. The expansion factors that weighted the analyses addressed these losses.

**Fig 3:**
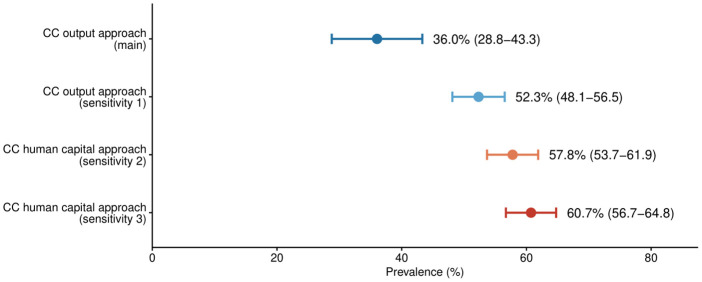
Prevalence and 95% confidence intervals of households of people with tuberculosis incurred with catastrophic costs (CC); First Guatemala national tuberculosis costs survey, 2023. Main: estimated by output approach with employment status before the onset of tuberculosis; Sensitivity 1: estimated by output approach with employment status at the interview; Sensitivity 2: estimated by human capital approach with employment status before the onset of tuberculosis; and Sensitivity 3: estimated by human capital approach with employment status at the interview.

The estimated catastrophic costs incurred by people with TB in Guatemala, through the output approach, was 36% (95% CI 29% − 43%). However, the sensitivity analysis adapted to the current employment status and the human capital approach indicated a burden exceeding 50% (Fig 3).

With regard to the sociodemographic characteristics of the study population, males constituted 52% (95% CI 45% − 59%), the age group of 15–44 years represented 45% (95% CI 37% − 52%), 53% (95% CI 46% − 61%) had completed primary school, 17% (95% CI 10% − 24%) was below the poverty line, 36% (95% CI 35% − 36%) from the South West region, and 53% (95% CI 49% − 57%) resided in households consisting of 0–4 people. The prevalence of reported social consequences was 75% (95% CI 72% − 79%), and 30% (95% CI 23% − 37%) of referred coping strategies were identified. As illustrated in Table 2, the distribution of these characteristics was further delineated according to the status of catastrophic costs.

**Table 2:** Sociodemographic characteristics of survey participants, according catastrophic costs status; First Guatemala national tuberculosis costs survey, 2023.

Variables		No catastrophic costs^a^		Catastrophic costs^a^		Overall	
		n	% (95% CI)	n	% (95% CI)	n	% (95% CI)
Sex	Female	170	49 (42 - 56)	79	47 (36 - 58)	249	48 (41 - 55)
	Male	166	51 (44 - 58)	115	53 (42 - 64)	281	52 (45 - 59)
Age group	0-14	76	21 (17 - 26)	18	9 (4 - 13)	94	17 (14 - 20)
	15-44	149	47 (39 - 54)	85	41 (31 - 50)	234	45 (37 - 52)
	45-64	86	24 (19 - 29)	63	38 (26 - 50)	149	29 (22 - 36)
	>65	25	8(5 - 12)	28	13 (8 - 18)	53	10 (7 - 13)
Educational level	No education	74	20 (16 - 25)	38	26 (12 - 40)	112	22 (15 - 29)
	Primary school	178	56 (49 - 63)	103	49 (38 - 59)	281	53 (46 - 61)
	Secondary school or higher	84	24 (18 - 29)	53	25 (18 - 33)	137	24 (21 - 28)
Employed before TB	No	8	3 (1 - 5)	4	2 (0 - 3)	12	2 (1 - 4)
	Yes	328	97 (95 - 99)	190	98 (97 - 100)	518	98 (96 - 99)
Sector of labor market	Formal	70	21 (16 - 26)	48	23 (16 - 30)	118	22 (18 - 25)
	Informal	266	79 (74 - 84)	146	77 (70 - 84)	412	78 (75 - 82)
Below poverty line	No	291	82 (73 - 90)	164	86 (81 - 91)	455	83 (76 - 90)
	Yes	45	18 (10 - 27)	30	14 (9 - 19)	75	17 (10 - 24)
Health insurance	No	274	81 (77 - 86)	159	77 (63 - 90)	433	80 (73 - 87)
	Yes	62	19 (14 - 23)	35	23 (10 - 37)	97	20 (13 - 27)
Region	Metropolitan (I)	67	18 (15 - 21)	48	23 (17 - 29)	115	20 (19 - 20)
	North (II)	10	6 (4 - 7)	2	2 (0 - 5)	12	4 (4 − 4)
	North East (III)	18	6 (4 - 8)	14	9 (5 - 12)	32	7 (7 − 7)
	South East (IV)	14	3 (2 - 4)	5	2 (0 - 4)	19	3 (3 − 3)
	Central (V)	91	19 (16 - 22)	71	26 (20 - 32)	162	22 (21 - 22)
	South West (VI)	132	41 (36 - 46)	51	27 (20 - 34)	183	36 (35 - 36)
	North West (VII)	1	5 (0 - 14)	1	9 (0 - 24)	2	6 (6 − 6)
	Peten (VIII)	3	2 (0 - 4)	2	3 (0 - 6)	5	2 (2 − 2)
Household size	0-4	179	50 (43 - 57)	128	59 (47 - 71)	307	53 (49 - 57)
	5+	157	50 (43 - 57)	66	41 (29 - 53)	223	47 (43 - 51)

^a^Catastrophic costs were defined when the percentage of total costs incurred by individuals treated for TB and their households that exceeds 20% of their annual household income.

TB: tuberculosis.

The prevalence of HIV positivity among the population was found to be 6% (95% CI 4% − 8%), while the prevalence of other comorbidities was found to be 36% (95% CI 29% − 43%). Furthermore, 7% (95% CI 5% − 10%) of the population had a history of TB, 81% (95% CI 77% − 84%) had bacteriologically confirmed pulmonary disease, and 20% (95% CI 13% − 26%) had a delayed diagnosis. Table 3 indicates that the treatment was carried out in primary health facilities in 89% of cases (95% CI 82% − 95%), and self-administered in 14% of cases (95% CI 11% − 16%).

**Table 3:** Clinical and treatment characteristics of survey participants, according catastrophic costs status; First Guatemala national tuberculosis costs survey, 2023.

Variables		No catastrophic costs^a^		Catastrophic costs^a^		Overall	
		n	% (95% CI)	n	% (95% CI)	n	% (95% CI)
HIV positivity	No	314	94 (92 - 97)	180	93 (90 - 97)	494	94 (92 - 96)
	Yes	22	6 (3 - 8)	14	7 (3 - 10)	36	6 (4 - 8)
Other comorbidities	No	231	71 (65 - 77)	108	52 (41 - 63)	339	64 (57 - 71)
	Yes	105	29 (23 - 35)	86	48 (37 - 59)	191	36 (29 - 43)
Previous tuberculosis	No	311	93 (90 - 96)	180	93 (89 - 97)	491	93 (90 - 95)
	Yes	25	7 (4 - 10)	14	7 (3 - 11)	39	7 (5 - 10)
DR-TB	No	330	98 (97 - 100)	192	99 (97 - 100)	522	99 (98 - 100)
	Yes	6	2 (0 - 3)	2	1 (0 - 3)	8	1 (0 - 2)
Type of tuberculosis	Bacteriologically confirmed pulmonary	258	78 (73 - 83)	166	85 (80 - 91)	424	81 (77 - 84)
	Clinically diagnosed pulmonary	63	18 (13 - 22)	18	9 (5 - 14)	81	15 (12 - 18)
	Extrapulmonary	15	4 (2 - 7)	10	5 (2 - 8)	25	5 (3 - 7)
Diagnosis delay	No	298	90 (87 - 93)	134	63 (51 - 76)	432	80 (74 - 87)
	Yes	38	10 (7 - 13)	60	37 (24 - 49)	98	20 (13 - 26)
Ever hospitalized	No	282	84 (80 - 88)	156	74 (60 - 87)	438	80 (73 - 87)
	Yes	54	16 (12 - 20)	38	26 (13 - 40)	92	20 (13 - 27)
Health facility type	Tertiary care	22	7 (4 - 11)	22	19 (4 - 33)	44	11 (5 - 18)
	Primary/secondary care	314	93 (89 - 96)	172	81 (67 - 96)	486	89 (82 - 95)
Treatment phase	Continuation	282	85 (82 - 89)	111	53 (42 - 64)	393	74 (67 - 81)
	Intensive	54	15 (11 - 18)	83	47 (36 - 58)	137	26 (19 - 33)
Self-administrated treatment	No	285	85 (81 - 89)	174	89 (85 - 94)	459	86 (84 - 89)
	Yes	51	15 (11 - 19)	20	11 (6 - 15)	71	14 (11 - 16)
Any social effect	No	103	30 (24 - 35)	35	17 (11 - 23)	138	25 (21 - 28)
	Yes	233	70 (65 - 76)	159	83 (77 - 89)	392	75 (72 - 79)
Coping strategies	No	243	69 (61 - 77)	136	73 (66 - 81)	379	70 (63 - 77)
	Yes	93	31 (23 - 39)	58	27 (19 - 34)	151	30 (23 - 37)

^a^Catastrophic costs were defined when the percentage of total costs incurred by individuals treated for TB and their households that exceeds 20% of their annual household income.

DR-TB: drug-resistant tuberculosis.

**Fig 4:**
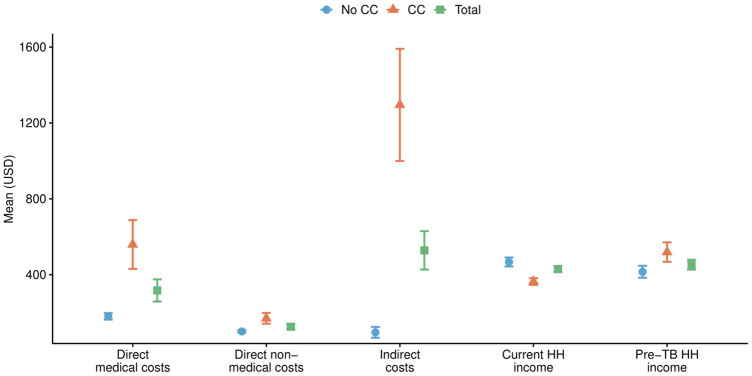
Means and 95% confidence intervals of income and incurred costs of households of people with tuberculosis, according catastrophic costs status; First Guatemala national tuberculosis costs survey, 2023.

The overall mean of the monthly household income was US$ 452.9 (95% CI 426.7–479.1) prior to TB diagnosis and US$429.9 (95% CI 414.7–445.1) at the time of the interview (Fig 4).

The means for direct medical, direct non-medical and indirect costs were US$ 317.1 (95% CI 258.7–375.5), US$ 125.9 (95% CI 112.4–139.4) and US$ 528.4 (95% CI 427.2–629.5), respectively (Table 4). With regard to proportionality, the mean percentage of total costs accounted for by direct medical costs was 32%, direct non-medical costs 13%, and indirect costs 55%.

**Table 4:** Means and 95% confidence intervals (95%CI) of incurred costs of households of people with tuberculosis, according drug sensitivity status; First Guatemala national tuberculosis costs survey, 2023.

Costs (US$)	Overall		Drug sensitive TB		DR TB	
	Mean	95% CI	Mean	95% CI	Mean	95% CI
**Total costs**	971.32	842.45–1,100.2	978.1	847.5–1,108.7	497.9	265.82–729.9
**Direct medical costs**	317.1	258.72–375.5	318.3	259.1–377.4	23.3	131.78–336.9
**Direct non-medical costs**	125.9	112.36–139.4	127.2	113.5–140.8	32.9	21.93–43.8
**Indirect costs**	528.4	427.25–629.5	532.6	430.1–635.1	230.7	0–499.2

**Fig 5:**
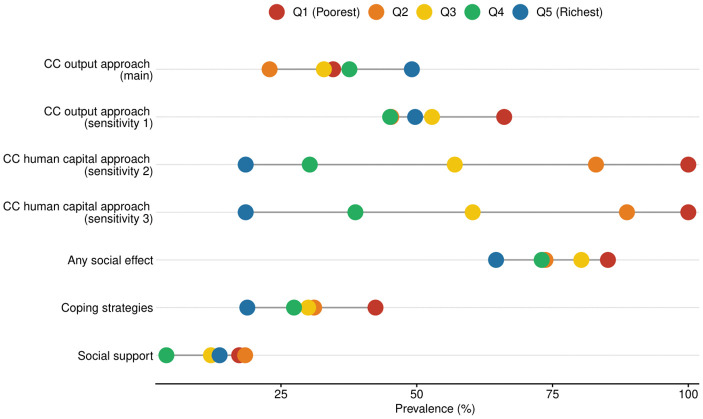
Prevalence of households of people with tuberculosis incurred with catastrophic costs, social effects, coping strategies and social support, according wealth quintiles; First Guatemala national tuberculosis costs survey, 2023. Main: estimated by output approach with employment status before the onset of tuberculosis; Sensitivity 1: estimated by output approach with employment status at the interview; Sensitivity 2: estimated by human capital approach with employment status before the onset of tuberculosis; and Sensitivity 3: estimated by human capital approach with employment status at the interview.

Fig 5 presents the catastrophic costs indicators and social support, any social effect and coping strategies variables according to wealth quintiles. The prevalence of catastrophic costs for the poorest quintile was 34.6%, compared with 49.1% for the richest quintile. Nevertheless, the output approach indicator of the sensitivity analysis demonstrated that the prevalence among the poorest quintile was 66.1%, while among the richest it was 49.7%, indicating an absolute inequality of 16.4%. Moreover, the disparities in prevalence among the wealth quintiles were accentuated when the human capital approach indicators were considered.

With regard to the analysis of associations, the prevalence of catastrophic costs was found to be higher among individuals aged between 15 and 44 years (PR 1.99; 95% CI 1.41–2.81). In contrast, the prevalence was lower for individuals from the South West region (PR 0.72; 95% CI 0.55–0.94), those residing in households comprising five or more individuals (PR 0.73; 95% CI 0.57–0.93), and those who underwent treatment in primary care health facilities (PR 0.69; 95% CI 0.49–0.97), as illustrated in Table 5.

**Table 5:** Adjusted^a^ prevalence ratios of hierarchical Poisson regression models of the association of sociodemographic and clinical characteristics of survey participants with catastrophic costs status; First Guatemala national tuberculosis costs survey, 2023.

Level	Characteristics		Prevalence ratio^b^	95% CI	p-value
1	Sex	Male	1.01	(0.80 - 1.28)	0.907
	Age group	15-44	1.99	(1.41 - 2.81)	<0.001
		45-64	0.74	(0.54–1.00)	0.051
		65+	1.07	(0.86 - 1.32)	0.553
	Educational level	Primary school	1.02	(0.77 - 1.33)	0.912
		Secondary school or higher	1.11	(0.91 - 1.35)	0.297
	Employed before TB	Yes	1.50	(0.62 - 3.65)	0.370
	Below poverty line	Yes	1.08	(0.75 - 1.56)	0.686
	Region	Metropolitan (I)	0.98	(0.72 - 1.32)	0.878
		North (II)	0.46	(0.14 - 1.49)	0.194
		North East (III)	0.97	(0.60 - 1.57)	0.909
		South East (IV)	0.58	(0.29 - 1.16)	0.122
		South West (VI)	0.74	(0.56 - 0.98)	0.036
		North West (VII)	1.00	(0.30 - 3.26)	0.994
		Peten (VIII)	1.00	(0.30 - 3.31)	0.999
	Household size	5+	0.76	(0.59 - 0.98)	0.033
	Health insurance	Yes	0.93	(0.71 - 1.23)	0.619
2	HIV positivity	Yes	0.88	(0.59 - 1.32)	0.545
	Other comorbidities	Yes	1.24	(0.97 - 1.6)	0.091
	Previous tuberculosis	Yes	1.03	(0.67 - 1.6)	0.891
3	DR-TB	Yes	0.71	(0.20 - 2.54)	0.601
	Type of tuberculosis	Clinically diagnosed pulmonary	1.05	(0.63 - 1.75)	0.848
		Extrapulmonary	0.91	(0.51 - 1.64)	0.752
	Diagnosis delay	Yes	0.93	(0.66 - 1.32)	0.691
	Ever hospitalized	Yes	1.19	(0.88 - 1.61)	0.266
	Health facility type	Primary/secondary care	0.69	(0.49 - 0.97)	0.035
	Self-administrated treatment	Yes	0.70	(0.45 - 1.10)	0.122
4	Social support	Yes	1.34	(0.91 - 1.98)	0.144

^a^The effect of each characteristic on the catastrophic costs is interpreted as having been adjusted for all the variables that belong to the hierarchical levels above it, as well as for those on the same level.

^b^Categories of reference: sex (female); age group (0–14 years old); educational level (no education); employment before (no); below poverty line (no); region (Central); household size (0–4); insurance (no); HIV positivity (no); other comorbidities (no); previous tuberculosis (no); DR-TB (no); tuberculosis type (bacteriologically confirmed pulmonary); diagnosis delay (no); ever hospitalized (no); facility type (tertiary care); self-administrated treatment (no); and social support (no).

DR-TB: drug-resistant tuberculosis; TB: tuberculosis.

Social effects (Table 6) were found to be more prevalent among individuals from the North West (PR 1.29; 95% CI 1.08–1.53) and North East regions (PR 1.21; 95% CI 1.00–1.46), and among those experiencing catastrophic costs (PR 1.21; 95% CI 1.09–1.34). Conversely, they were observed to be lower for the 45–64 age group (PR 0.77; 95% CI 0.67–0.89), for individuals with primary school education (PR 0.83; 95% CI 0.73–0.96), and for households comprising five or more residents (PR 0.86; 95% CI 0.77–0.96).

**Table 6:** Adjusted^a^ prevalence ratios of hierarchical Poisson regression models of the association of sociodemographic and clinical characteristics of survey participants with the presence of any social effect and coping strategies; First Guatemala national tuberculosis costs survey, 2023.

Level	Characteristics		Any social effect			Coping strategies		
			PR^b^	95% CI	p-value	PR^b^	95% CI	p-value
1	Sex	Male	0.94	(0.85 - 1.03)	0.174	1.03	(0.97 - 1.09)	0.396
	Age group	15-44	1.09	(0.92 - 1.29)	0.334	0.91	(0.83 - 0.98)	0.016
		45-64	0.77	(0.67 - 0.89)	0.001	1.00	(0.93 - 1.08)	0.983
		65+	1.04	(0.95 - 1.14)	0.381	0.96	(0.91 - 1.02)	0.199
	Educational level	Primary school	0.83	(0.73 - 0.96)	0.011	1.06	(0.98 - 1.13)	0.126
		Secondary school	0.94	(0.86 - 1.03)	0.174	0.92	(0.88 - 0.97)	0.002
	Employed before TB	Yes	1.17	(0.82 - 1.67)	0.390	1.16	(0.92 - 1.47)	0.201
	Below poverty line	Yes	1.12	(1.00 - 1.27)	0.055	1.11	(1.02 - 1.19)	0.010
	Region	Metropolitan (I)	1.08	(0.94 - 1.25)	0.265	0.98	(0.90 - 1.06)	0.574
		North (II)	1.16	(0.96 - 1.4)	0.133	0.83	(0.72 - 0.96)	0.015
		North East (III)	1.21	(1.00 - 1.46)	0.050	1.09	(0.95 - 1.25)	0.238
		South East (IV)	1.10	(0.87 - 1.39)	0.408	1.26	(1.09 - 1.46)	0.002
		South West (VI)	0.96	(0.84 - 1.09)	0.500	1.02	(0.95 - 1.10)	0.630
		North West (VII)	1.29	(1.08 - 1.53)	0.005	1.28	(0.98 - 1.66)	0.065
		Peten (VIII)	0.28	(0.03 - 2.2)	0.223	1.14	(0.86 - 1.52)	0.354
	Household size	5+	0.86	(0.77 - 0.96)	0.007	0.90	(0.85 - 0.96)	0.002
	Health insurance	Yes	0.96	(0.82 - 1.12)	0.589	0.95	(0.88 - 1.02)	0.154
2	HIV positivity	Yes	0.89	(0.72 - 1.09)	0.252	1.01	(0.90 - 1.14)	0.867
	Other comorbidities	Yes	0.91	(0.82 - 1.02)	0.107	1.06	(0.99 - 1.14)	0.104
	Previous tuberculosis	Yes	0.93	(0.77 - 1.13)	0.459	1.00	(0.89 - 1.13)	0.989
3	DR-TB	Yes	0.79	(0.47 - 1.31)	0.357	0.88	(0.69 - 1.12)	0.300
	Type of tuberculosis	Clinically diagnosed pulmonary	0.95	(0.75 - 1.19)	0.636	1.13	(1.02 - 1.25)	0.017
		Extrapulmonary	1.08	(0.86 - 1.37)	0.501	1.16	(1.01 - 1.33)	0.033
	Diagnosis delay	Yes	1.32	(0.98 - 1.77)	0.063	1.07	(0.96 - 1.20)	0.222
	Ever hospitalized	Yes	1.05	(0.94 - 1.19)	0.384	1.02	(0.94 - 1.10)	0.710
	Health facility type	Primary/secondary care	1.16	(0.97 - 1.4)	0.108	1.06	(0.95 - 1.18)	0.296
	Self-administrated treatment	Yes	1.16	(0.99 - 1.36)	0.076	1.09	(0.99 - 1.20)	0.071
4	Social support	Yes	1.09	(0.94 - 1.26)	0.254	1.11	(1.01 - 1.22)	0.024
	Catastrophic costs	Yes	1.21	(1.09 - 1.34)	<0.001	1.00	(0.94 - 1.07)	0.970

^a^The effect of each characteristic on the catastrophic costs is interpreted as having been adjusted for all the variables that belong to the hierarchical levels above it, as well as for those on the same level.

^b^Categories of reference: sex (female); age group (0–14 years old); educational level (no education); employment before (no); below poverty line (no); region (Central); household size (0–4); insurance (no); HIV positivity (no); other comorbidities (no); previous tuberculosis (no); DR-TB (no); tuberculosis type (bacteriologically confirmed pulmonary); diagnosis delay (no); ever hospitalized (no); facility type (tertiary care); self-administrated treatment (no); social support (no); and catastrophic costs (no).

PR: prevalence ratio; DR-TB: drug-resistant tuberculosis; TB: tuberculosis.

The utilization of coping strategies was found to be more prevalent among individuals below the poverty line (PR 1.11; 95% CI 1.02–1.19) and those originating from the South East region (PR 1.26; 95% CI 1.09–1.46). The analysis also revealed that clinically diagnosed pulmonary TB (PR 1.13; 95% CI 1.02–1.25), extrapulmonary TB (PR 1.16; 95% CI 1.01–1.33) and the presence of social support (PR 1.11; 95% CI 1.01–1.22) were associated with increased utilization of coping strategies. A reduced use of coping strategies was identified among people aged 15−44 years (PR 0.91; 95% CI 0.83–0.98), those with secondary school or higher education (PR 0.92; 95% CI 0.88–0.97), those residing in the North region (PR 0.83; 95% CI 0.72–0.96), and those living in households with five or more residents (PR 0.90; 95% CI 0.85–0.96).

## Discussion

In 2023, approximately 40% of individuals receiving treatment for TB incurred catastrophic costs in Guatemala, according to the main output approach. However, if the sensitivity analysis is considered, this number can reach six out of ten. The study identified inequalities in the occurrence of catastrophic costs, social effects and coping across wealth quintiles. Catastrophic costs were observed to be twice as high among individuals aged between 15 and 44 years. However, these costs were found to be lower among individuals from the South West, households with five or more individuals, and those who underwent treatment in primary/secondary facilities. Geographic region and catastrophic costs increased the prevalence of social effects, while the 45–64 age group, primary school education and households with five or more residents decreased it. The employment of coping strategies was found to be associated with sociodemographic characteristics, as well as clinically diagnosed pulmonary and extrapulmonary TB.

This was the firstly conducted national study, endorsed by the WHO, to estimate the burden of the catastrophic costs incurred by households of people with TB in Guatemala. The utilization of standardized and previously validated methods, the incorporation of sensitivity analyses and the adoption of regression models based on a theoretical model are further strengths of the study. Analyses employing both the output and human capital approaches offer advantages and disadvantages. Each of these metrics focuses on a distinct aspect of indirect costs, yet none offers a comprehensive assessment [9]. Therefore, both were presented and should be interpreted in a complementary manner to provide a broad representation of the TB catastrophic costs burden in the country, irrespective of whether the primary or sensitivity approach is employed. Furthermore, adjustment for current employment status was considered essential to avoid underestimations and enhance metrics even further. Finally, the incorporation of specific models for social effects and coping strategies contributed to a more comprehensive view of the potential implications of TB treatment.

However, it is acknowledged that limitations were also present and should be accounted for. Among the limitations of the study are the self-reported data collection, which could lead to recall bias, the cross-sectional strategy to collect longitudinal data, and the data extrapolations. Nevertheless, the utilization of standardized methods and the existence of a preceding body of research serve to reinforce the validity of the approach and the robustness of the results. Although participants in the intensified and continuation phases were not sampled using a stratified approach, the final sample balanced the two groups and reflected the treatment distribution and previous publications [7,13]. In settings characterized by a predominance of informal workforce participation, such as Guatemala, the reliability of self-reported income data can be compromised. However, when income was estimated based on household characteristics, the values were close to self-reported income, thereby reinforcing the robustness of the results. As the study approvals and data collection were conducted prior to the publication of the revised recommendations (Consolidated guidance on tuberculosis data generation and use. Module 4: Surveys of costs faced by households affected by tuberculosis), the income loss (indirect costs) did not encompass the costs from the onset of symptoms to the diagnosis [9]. This may potentially result in an underestimation of the indicators, despite being consistent with previous publications. Missing data was handled using component-specific strategies to enhance feasibility [9], but this may introduce bias if the joint distribution between cost components and household income is not preserved. Finally, it is important to note that the catastrophic costs indicators presented did not consider people experiencing homelessness or those subjected to circumstances of deprivation of liberty.

A meta-regression analysis, conducted to estimate the overall burden of catastrophic costs for households of individuals diagnosed with TB in low- and middle-income countries, indicated an estimated burden of 54.9% (uncertainty level 47.0% − 63.2%), with a higher estimate of 64.7% (uncertainty level 56.4% − 73.3%) observed for countries in the region of the Americas [5]. The findings of this study exceed the primary results of the current research, which is the indicator monitored by the WHO to evaluate the progress of the country towards End TB target. However, the sensitivity analysis, which considers the employment status at the time of the interview to estimate indirect costs, suggests an underestimation of this measure in the main analysis and, consequently, an underestimation of the indicator. This can be explained by the variability of 68 percentage points in the employment status of the participants, with only 30% of them employed at the time of the interview. Consequently, it is hypothesized that the actual TB catastrophic cost burden in Guatemala is aligned with the sensitivity analysis and meta-regression, thus placing it among the countries with the highest costs.

These results underscore the urgent need for multisectoral action to address the financial burden of TB in Guatemala, in line with the End TB Strategy target of eliminating catastrophic costs by 2030. In light of the high proportion of employment status changes that occurred during TB treatment, collaboration between the Tuberculosis Program and the Ministries of Labor and Social Security, along with other relevant actors, could be envisaged as one of these actions. Such collaboration could facilitate the development of a plan that prioritizes the protection of the employment of people with TB during their treatment. This plan could be anchored by the MDG Acceleration Framework methodology and the Guidance on social protection for people affected by tuberculosis as potential approaches [14,15]. In Guatemala, the governmental social protection network comprises conditional cash transfer programs, direct food assistance, and youth inclusion and development programs [16]. The Bono Social constitutes a program of conditional cash transfers whose objective is to reduce poverty and encourage the use of health and education services [16]. The Bolsa Social is another program that offers food assistance in the form of conditional cash transfers for the purchase of basic food items [16]. The Comedores Sociales is a network of public dining halls that ensures access to basic food [16]. These programs have the potential to alleviate the financial burden of TB. Thus, the previously suggested initiatives could optimize access to these programs for people with TB.

With respect to the total costs incurred in the care of TB, the aforementioned study also found an average total cost of US$1,253 per TB episode [5], a finding that is corroborated by another systematic review and meta-analysis which found an average weighted total cost for DS-TB of US$1,259 [17]. Both figures exceeded the results obtained from the Guatemala study. However, it should be noted that the World Bank estimates that, in 2024, 57.3% of Guatemalans live in poverty, with an average income of $6.85 per day [8]. This is a context which imposes significant hardship on the population, and it is hypothesized that the lower expenditure could be related to the absence of resources. An additional noteworthy observation pertains to the cost breakdown analysis, wherein the proportion of medical costs in this study was found to be surpassed only by that of Mali (41%), a finding that aligns with the observed pattern of high out-of-pocket health expenditure in the country [6,18].

One potential strategy for reducing direct medical costs, which can be introduced promptly by health services, is the implementation or reinforcement of active case finding. This approach has been demonstrated to result in a reduction in expenditure associated with diagnostic seeking [17], despite the findings of this study suggest a lack of association between delayed diagnosis and catastrophic costs. In Guatemala, measures such as the scaling up of specialized tests in the public sector and the optimization of processes for referring individuals from tertiary care are potentially effective in reducing direct medical and non-medical costs, respectively. Other strategies that services could adopt, but with effect on indirect costs, include the organization of care so that different activities (consultations, collection of medicines, tests, etc.) are carried out concurrently, the alignment of appointments with the working routines of people undergoing treatment, and the expansion of strategies that use digital technologies, such as treatment directly observed by video. These measures have the potential to reduce expenditure on transport, food and potential loss of work hours; however, it is important to note that they do not exclude the necessity of planning more audacious strategies, preferably in partnership with sectors outside the health field, such as offering nutritional support, transportation subsidies and guaranteed minimum wage maintenance.

In Guatemala, while the poorest quintiles demonstrated a reduced burden in the primary indicator, the sensitivity analysis revealed an inversion in this situation. This finding suggests that the adjustments made to the primary indicator are appropriate for the local context, which was characterized by a high proportion of individuals engaged in informal employment and notable changes in employment status. The primary indicator derived from the human capital approach for the country should therefore be analyzed with caution, given the possibility of a differential measurement error regarding indirect costs and a consequent misclassification of catastrophic costs [19]. Assessments conducted in similar scenarios, characterized by high labor insecurity, should incorporate corresponding adjustments to properly address the situation.

Consequently, these results suggest that the poorest population were subject to the greatest catastrophic costs and social effects, and this same group had a reduced use of coping strategies, which, due to the limitations of the data, precludes confirmation of whether this is attributable to an absence of need or access to coping strategies. This situation served to reinforce the inequalities of these distributions. The same pattern was identified in previous studies, where wealthier quintiles were less affected by catastrophic costs [5,20]. However, it is important to note that even in surveys which identified higher expenditure on TB treatment among wealthier groups, such as in Bangladesh [21], this did not necessarily indicate a higher burden on the proportion of the income affected. In China, a cross-sectional study based on the National Tuberculosis Cost Survey, conducted in 2017, revealed that while there was equality in costs due to pre-treatment and treatment care, there was inequality in the distribution of catastrophic costs, which was also observed across various population sub-groups [22]. Since these inequalities were socially produced (and therefore modifiable) and can be defined as unfair, it is also pivotal to be aware of the inequities in catastrophic costs occurrence [23].

Regarding the factors associated with catastrophic costs, the prevalence was twice as high among the economically active age group, which is in line with the characteristics of the country, including low job quality and a high participation level in the informal sector, resulting in a greater number of vulnerable workers [8]. The South West region, which is characterized by a rural profile and a pronounced indigenous population [24], as well as households comprising five or more individuals, where a greater number of people could contribute to the total income, evidenced a lower propensity to incur catastrophic costs, in line with findings from Philippines [25]. Furthermore, the reduced incidence of catastrophic costs among individuals treated in primary and secondary facilities can be attributed to the lower complexity of cases treated in these units, along with their more widespread distribution [26], in contrast to tertiary facilities.

A higher prevalence of social effects was observed among individuals residing in the North West and North East regions. The regions in question are distinguished by ethnic diversity, inequality in access to education and basic services in the North West, and an increased indigenous presence, extreme poverty, low access to basic services and education in the North East [24]. Those experiencing catastrophic costs also presented a higher probability of social effects. In the Colombian context, the adjusted odds ratio for catastrophic costs was 2.1 (95% CI 1.5–3.0) for those who experienced job loss, in comparison with those who did not [4]. In contrast, households comprising individuals from older age groups, with a higher level of education, and with five or more residents, showed a reduced prevalence of social effects, which can be accounted for by the presence of a more stable lifestyle.

Finally, in this study, the occurrence of catastrophic costs was not associated with the utilization of coping strategies, which may be more indicative of an absence of coping mechanisms than a lack of necessity. Another study, using data from India, Bangladesh and Tanzania, aimed to determine whether dissaving (the sale of assets or uptake of loans) could be used as an indicator of financial hardship [27]. It suggested that dissaving could potentially serve as a convenient proxy for catastrophic costs, avoiding the need for complex cost questionnaires [27], which doesn’t seem to apply to the Guatemalan context. Additionally, a prospective cohort study of 257 participants in Egypt, in 2019, also found that coping strategies were associated with catastrophic costs (adjusted OR 4.87; 95% CI 3.19–10.84) [28].

## Conclusion

The baseline estimative of the catastrophic costs of TB in Guatemala suggested that four to six out of ten individuals receiving treatment incurred catastrophic costs, a burden marked by inequities across wealth quintiles, challenging the country to achieve zero catastrophic costs of TB.

However, the analyses also pinpointed some pathways that could be followed when building policies to effectively reduce the burden of the catastrophic costs. Notwithstanding the fact that TB treatment is provided free of charge, the Tuberculosis Program could collaborate with other sectors of the Ministry of Health and Social Assistance of Guatemala to expand policies for the free treatment of TB-related comorbidities. Such collaboration could focus on the following areas: diagnostic tests; follow-up laboratory tests; specific clinical or laboratory assessments; and the decentralization of care. Comprehensive social protection strategies addressing people with TB, which may combine support for transport, food, subsidies, and cash transfers, could also be developed based on the MDG Acceleration Framework and the Guidance on social protection for people affected by tuberculosis. In instances where prioritization is indicated, a systematic approach guided by specific criteria, such as the regions of the country, the type of facility where treatment is performed, and some individual and household characteristics, must be employed. Additionally, since the necessary measures extend beyond the health sector, it is essential to include representatives from different government sectors, as well as non-governmental and civil society organizations, in the formulation of these policies.

Achieving zero catastrophic costs for TB in Guatemala is a long-term goal, but this study is a solid first step. We look forward to seeing it develop into practical initiatives, such as those suggested here, to reduce TB treatment costs and the burden of catastrophic costs in the country.
