# Supplementary material for: Burden of catastrophic costs, income inequalities and associated factors in Guatemala: First national tuberculosis costs survey, 2023
Source: PLoS One. 2026 Aug 17;21(8):e0351164. doi: 10.1371/journal.pone.0351164 (PMC13480651; doi:10.1371/journal.pone.0351164)
Supplement: Supplementary file 1 [file pone.0351164.s001.docx]

Thank you for the opportunity to review this manuscript. The co-authors should be commended for strong writing and for producing Guatemala’s first national TB patient cost survey. The manuscript addresses an important evidence gap in the region, and the analysis is timely and policy-relevant.

Below, I outline suggestions that may strengthen clarity, methodological transparency, and interpretive depth.

**Abstract**

Consider defining *catastrophic costs* directly in the abstract to improve accessibility for readers less familiar with TB cost-survey methodologies

**Introduction**

Tuberculosis is written out in most instances; however, the accepted abbreviation **TB** could be used more consistently to improve readability.

Third paragraph of the Introduction should be copy edited. You are missing a parenthesis after 48%. The phrase “actual situation” could be replaced with more precise scientific language. The last sentence of the paragraph repeats the word strategies and could be streamlined for clarity.

**Methods**

The authors should justify why the output approach was selected as the main method for estimating indirect costs. The WHO handbook recommends its use in predominantly *formal* economies, yet the study population shows a high level of informal employment. Additional explanation is needed to clarify why this approach is most appropriate in this setting.

I would suggest that the Measurements section should precede Variables, as the current order left several variable definitions unclear during initial reading. If the authors prefer, they could also create a box for the definition of variables.The authos should provide clearer definitions for ‘ever hospitalized’. Does this mean during the episode of TB or throughout the person’s life? “Social support” includes what types? Define Any social effect should state the specific items included.

The section labeled *Bias* describes steps taken to reduce bias, not the sources of bias themselves. Consider renaming it to Bias Handling, Mitigation of Bias, or a similar term.

Quality control of data entry should be better explained.

The pilot study is briefly mentioned but could be described more fully (e.g., sample used, changes made following the pilot).

The study does not provide sufficient information about the selection of clusters. More information is needed on how clusters were distributed across regions and facilities. The sample size calculation specifies 44 clusters, yet only 42 were used. Clarification is needed on why two clusters were not included and whether this affects representativeness.

The description of extrapolation methods in the Statistical Methods section should be expanded to ensure transparency.

Chart 1 indicates rules for “Employed before onset of TB,” but the accompanying text suggests these rules apply to income before and during treatment. This requires clarification. Please provide a rationale for why missing income values were replaced with zero under the output approach, yet estimated using average wages or minimum wages under the human capital approach. It may introduce systematic bias.

In addition to missing values, the manuscript should clarify how zero income was handled analytically.

**Results**

The results are clearly presented; figures and tables are aligned with WHO reporting.

The eight development regions are currently listed alphabetically. Guatemala typically numbers its regions; arranging them in numeric or geographic order may improve interpretability.

The reversal of the wealth gradient between main and sensitivity analyses is particularly important but is treated briefly.

Some regions (e.g., Petén, North West) have very small denominators (1–5 participants), raising concerns about the stability of prevalence ratio estimates.

The hierarchical regression framework is appropriate, but the conceptual model used to assign variables to levels is not sufficiently described. The linkage between the theoretical model in Methods and Tables 3 & 4 should be strengthened.

The manuscript refers to “access” to coping strategies. While access to credit may be constrained, the sale of assets is generally not an issue of access. The authors may wish to reconsider this terminology and provide a full definition of the coping strategies assessed.

Citation 13 appears to be incomplete; I was unable to retrieve it with the information provided

**Discussion**

The Discussion synthesizes findings well and situates Guatemala within global patterns of catastrophic costs. Policy recommendations are aligned with WHO guidance.

The interpretation of the large discrepancies observed between the main and sensitivity analyses could be expanded.

Greater attention could be given to Guatemala’s health-system context, particularly its extremely high out-of-pocket expenditure, fragmented and underfunded public services, and limited, uneven social assistance coverage. The manuscript could also elaborate on health financing structures in Guatemala, providing context for why TB-affected households face high direct medical and non-medical costs.

Recommendations related to social protection could be more concrete (e.g., transport subsidies, nutritional support, labor protections, integration with existing cash-transfer schemes).

The authors should consider incorporating critiques of income-based approaches to estimating financial hardship. For example, the following article offers relevant conceptual framing:
*Timire et al., Clinical Infectious Diseases (2023), “Use of a Sustainable Livelihood Framework–Based Measure to Estimate Socioeconomic Impact of TB on Households.”*

While the suggestions to reduce medical costs through active case finding, streamlining service delivery, and VOT are important, the Discussion could additionally highlight broader social-protection measures capable of reducing both direct and indirect costs.
