# Supplementary material for: Burden of catastrophic costs, income inequalities and associated factors in Guatemala: First national tuberculosis costs survey, 2023
Source: PLoS One. 2026 Aug 17;21(8):e0351164. doi: 10.1371/journal.pone.0351164 (PMC13480651; doi:10.1371/journal.pone.0351164)
Supplement: Supplementary file 2 [file pone.0351164.s003.docx]

**Overall Assessment**

This is a strong and policy-relevant manuscript presenting Guatemala’s first national tuberculosis (TB) patient cost survey. It addresses an important evidence gap in the Americas and provides timely data on catastrophic costs, inequalities, and associated factors. The analysis is broadly sound and aligned with WHO guidance, and the findings have clear implications for financial protection policies.

With revisions to improve clarity, consistency, and interpretive depth, the manuscript will be well positioned for publication.

**Major Comments**

1. **Methods**

- While the sampling framework (cluster design, ICC, regional stratification) is described well, participant selection within facilities remains unclear.
- Please clarify how participants were recruited at the facility level and how representation across intensive vs. continuation phases was achieved.
- The handling of missing and zero income values differs across approaches (e.g., replacement with zero vs. imputed wages), which may introduce systematic bias in the estimation of indirect costs. This warrants clearer justification and discussion of potential implications.
- The description of cost extrapolation across treatment phases would benefit from clearer explanation within the statistical methods.
- The variable “social support” is included in the analysis but not clearly defined. Please specify what constitutes social support. Similarly, ensure that all composite variables (e.g., social effects, coping strategies) are clearly and consistently defined.

2. **Results**

- Consider including an additional table presenting disaggregated costs (direct medical, direct non-medical, indirect costs), which would improve interpretability and align with standard TB cost survey reporting.

3. **Discussion**

- The Discussion would benefit from a clearer structure, with the key findings from Guatemala presented and interpreted upfront, before moving to strengths and limitations.
- The manuscript engages with the updated WHO guidance recommending the output approach as the primary measure and makes a clear effort to present complementary analyses. However, this important methodological tension could be more explicitly foregrounded and analytically developed. In particular, the reversal of the wealth gradient between the main and sensitivity analyses is a central finding that warrants deeper interpretation, rather than primarily methodological explanation. The Discussion would benefit from more clearly articulating what this divergence implies for the validity of the primary measure in Guatemala’s context of high labor informality, and from taking a more explicit position on how WHO-recommended approaches perform in such settings. Strengthening this interpretation would enhance both the scientific contribution and the policy relevance of the manuscript.
- The Discussion could also be strengthened by engaging with critiques of income-based measures of financial hardship. For example, work using a sustainable livelihoods framework (e.g., Timire et al., 2023) may provide a useful starting point.
- Finally, increased engagement with the WHO guidance on social protection for TB-affected people and the Multisectoral Accountability Framework would further strengthen the policy framing around potential strategies to reduce catastrophic costs.

**Minor Comments**

4. **Abstract**

- The abstract could be strengthened by presenting results more quantitatively, particularly for associated factors, social effects, and coping strategies.

5. **Introduction**

- The statement that TB cost survey data have informed policy could be strengthened by referencing broader global examples. Fo example, see: <https://www.ncbi.nlm.nih.gov/pmc/articles/PMC6645445/>

6. **Consistency**

- Standardize references to the Ministry of Health (currently appears as “Ministry of Health,” “Guatemala Ministry of Health,” and “Ministry of Health and Social Assistance”).
- Figure numbering and sequencing should be carefully checked, as there appear to be inconsistencies.
- The manuscript uses inconsistent numbers of decimal places across tables and text; these should be standardized to improve clarity and consistency.

7. **Justification of exclusion criteria**

- The exclusion of individuals experiencing homelessness should be briefly justified, given their likely vulnerability to catastrophic costs.

8. **Redundancy and repetition**

- Survey dates are repeated multiple times across sections.
- The paragraph following Chart 1 is repetitive and could be condensed.
- The hierarchical frameworks across outcomes are very similar and could potentially be streamlined.

9. **Language and style**

- Additional copyediting is needed to address minor grammatical issues and inconsistencies.
- Contractions in the Discussion should be removed to maintain a formal tone.
- The final paragraph of the Conclusions differs stylistically and should be aligned with the rest of the manuscript.
