# Supplementary material for: Burden of catastrophic costs, income inequalities and associated factors in Guatemala: First national tuberculosis costs survey, 2023
Source: PLoS One. 2026 Aug 17;21(8):e0351164. doi: 10.1371/journal.pone.0351164 (PMC13480651; doi:10.1371/journal.pone.0351164)
Supplement: Supplementary file 3 [file pone.0351164.s004.docx]

Response to reviewers

Dear Editor and Reviewers,

We are grateful for all the comments and contributions that have been provided, which have undoubtedly contributed to the enhancement of our manuscript.

We have attempted to address all the suggestions made.

In the following section, we provide a comprehensive response to each of the aforementioned comments.

***Editor’s comments:***

*Across both reviews, the most critical issues related to methodological transparency, internal consistency, and interpretation. In particular, please:

1. Clarify and justify methodological choices, especially the use of the output approach as the primary method for estimating indirect costs in a setting with a large informal economy, given WHO guidance. Expand the description of key equations, assumptions, handling of zero and missing income values, extrapolation methods, and the rationale for differences between the output and human capital approaches.*
**Author’s response: With regard to indicators, the WHO guide published in 2017 [1] actually advised against the use of the output approach for scenarios with a high proportion of informal labor market. However, the most recent publication in 2025 [2] has introduced a significant alteration to this approach. The new guideline advocates for analyses to encompass both approaches, with output designated as the primary indicator and human capital used for sensitivity analysis [2]. This shift in recommendation is attributable to the findings highlighting diverse and complementary dimensions of the object of study. Furthermore, additional analyses were incorporated to address the change in employment status that occurred among the study participants. This information was elucidated in the text (lines: 389-392; 888-900).**

**Subsequent modifications were made to the text of the manuscript in order to ensure that a comprehensive description of the key equations (lines: 158-173; 352-353), assumptions (lines: 198-201; 336-337; 401-405; 418-423), handling of zero and missing income values (lines: 357-373), and extrapolation methods (lines: 350-356) was provided.**

*2. Strengthen reporting of the sampling design, including cluster selection, cluster size, ICC and design effect, regional distribution of clusters, and clarification of discrepancies between planned and achieved clusters. Provide precise study dates in the Methods and Results.*
**Author’s response: Further information has been provided on sample design, sampling process, and losses (lines: 336-337; 341-342; 346-349; 444-447). In addition, the precise dates of data collection were replicated in the different sections (lines: 111; 443).**

*3. Improve clarity and consistency in definitions and structure, including clearer variable definitions (e.g., hospitalization, social support, social effects, coping strategies), reordering or restructuring sections where helpful, and clearer linkage between the conceptual framework and regression models. Please also comment on potential over-adjustment in regression analyses given outcome prevalence.*
**Author’s response: The text has been restructured in accordance with the recommendations (lines: 139-231). The definitions of variables have undergone a revision (lines: 198-231). The rationale supporting the theoretical model and the defined hierarchical levels has also been incorporated (lines: 401-405), in addition to the discussion on potential overfitting (lines: 418-423).**

**In relation to the final topic, it is important to emphasize that the regression models implemented in the study were structured to minimize the probability of overadjustment. In spite of the cross-sectional nature of the study, it can be asserted that the independent variables included in the model precede catastrophic costs, which is an outcome of treatment [3]. Moreover, the hierarchical entry of independent variables into the model avoided adjustment by intermediate variables [3].**

*4. Address discrepancies between main and sensitivity analyses, particularly the reversal of the wealth gradient and unexpected findings such as higher catastrophic costs in richer quintiles. Ensure that the abstract reflects the main analysis rather than sensitivity analyses, and expand discussion of these differences.*
**Author’s response: The discussion related to this aspect has been improved (lines: 1002-1010), and all necessary information for correct interpretation has been included in the abstract (lines: 31-43).**

*5. Revise the Discussion for accuracy and alignment with results, avoiding claims not supported by the study (e.g., effects of active case finding or poorest quintiles bearing the highest burden), and more carefully situating Guatemala’s findings relative to global estimates. Additional contextualization of Guatemala’s health financing and social protection landscape would strengthen the policy relevance of your paper.*
**Author’s response: The discussion section has undergone extensive revision to ensure that all information presented is supported. The discussion of the findings (lines: 1002-1010; 923-935) and the contextualization of the estimates has been advanced (lines: 944-965).**

*6. Attend to presentation and completeness, including careful proofreading, correction of minor errors and missing citations, clearer figure/table explanations, consideration of a map of study facilities, and ensuring data availability in line with PLOS One requirements and reviewer comments.*

**Author’s response: The text, references and illustrations have been revised. The map has been incorporated in accordance with the recommendation. The data can be accessed via the link:** https://doi.org/10.6084/m9.figshare.31708996

***Reviewers' comments:***

*1. Is the manuscript technically sound, and do the data support the conclusions?
Reviewer #1: Yes*

*Reviewer #2: Partly*

**Author’s response: The manuscript has undergone a comprehensive revision to ensure that all information supporting the conclusions is explicitly described.**

*2. Has the statistical analysis been performed appropriately and rigorously?*

*Reviewer #1: Yes*

*Reviewer #2: No*

**Author’s response: The description of statistical methods was improved to ensure that all strategies adopted were detailed, as recommended by the reviewers.**

*3. Have the authors made all data underlying the findings in their manuscript fully available?*

*Reviewer #1: No*

*Reviewer #2: No*

**Author’s response: The data can be accessed via the link:** https://doi.org/10.6084/m9.figshare.31708996

*4. Is the manuscript presented in an intelligible fashion and written in standard English?*

*Reviewer #1: Yes*

*Reviewer #2: No*

**Author’s response: The manuscript has undergone extensive revision to ensure compliance with academic standards.**

*5. Review Comments to the Author
Reviewer #1: The authors have written that they will make their data available once the manuscript has been approved, but at the present moment, the manuscript does not align with PLOS One's Data Policy. The editor may need to request that the authors either provide full access to the dataset prior to further review or formally justify an exemption under PLOS ONE’s data-availability guidelines before the manuscript can proceed in the review process.*

**Author’s response: The data can be accessed via the link:** https://doi.org/10.6084/m9.figshare.31708996

*Reviewer #2: The authors present a study of patient costs among people with TB in Guatemala in 2023. The authors found 36% of participants experienced catastrophic costs, using the output approach. Catastrophic costs were more common among the youngest age groups. This is an important question and I commend the authors for seeking to contribute to a nascent literature in the Americas. There are likely important insights that can be gleaned from this work. I have several comments for improvement.*

**Author’s response: Thank you for your careful review and comments. We really appreciate the contributions they made to the improvement of the manuscript. We have attempted to address all the suggestions to the best of our ability.**

*1. Please report the cluster size and ICC used to calculate the design effect.*

**Author’s response: The passage was rewritten and the information was added to improve understanding (lines: 336-337).**

*2. Please provide precise dates of the study when you mention it. 2023 is fine for the title, but precise dates should be provided in the methods and results (I recognize it is provided once).*

**Author’s response: The precise dates on which the data were collected for the study were incorporated into both the design subsection (line: 111) and the presentation of results (line: 443). However, in the discussion section, the year (2023) was retained, given that the methods employed in the study enable the inference of results for that year.^1^**

*3. The abstract appears to report a sensitivity analysis by income quintile, rather than the main analysis. The numbers appear highly unlikely.*

**Author’s response: Indeed, the results presented are from the sensitivity analysis, and the methods do not provide adequate support for their interpretation. The text has been revised (lines: 32-43).**

*4. PLoS One is a general journal. It would be helpful to expand upon the methods and equations used to estimate key outcomes (e.g., equations used for the output approach, human capital approach). This is missing and would help make clear to readers why some unexpected findings (e.g., catastrophic costs largest in the richest quintile) occur.*

**Author’s response: The method for calculating the indicators had been defined in the variables’ subsection (lines: 153-168; 175-182). However, following the reviewer's request, the statistical methods were revisited and the information re-entered using figure with the equations [lines: 170 (Figure 1); 350-353].**

*5. I do not believe PLoS One provides copyediting. The manuscript should be proofread carefully and corrected for grammar.*

**Author’s response: We appreciate your comment. The manuscript has undergone extensive revision.**

*6. Readers may find it helpful to have a map of the included health facilities in Guatemala.*

**Author’s response: The map has been included and is presented as the new Figure 2 (line: 449).**

*7. The regression analyses may be over-adjusted based on the prevalence of the outcome, particularly for catastrophic costs. This should be commented on.*

**Author’s response: As recommended, a comment on this topic has been included in the methods (lines: 418-423; 893-900). The regression models implemented in the study were structured to minimize the probability of overadjustment. In spite of the cross-sectional nature of the study, it can be asserted that the independent variables included in the model precede catastrophic costs, which is an outcome of treatment.^2^ Moreover, the hierarchical entry of independent variables into the model avoided adjustment by intermediate variables.^2^**

*8. In the discussion, it is stated that the findings of this analysis are congruent with a global meta-regression. However, this is not the case for the study's main analysis. Indeed, the majority of studies informing the global meta-regression used the output approach, and so it appears Guatemala may have a much lower proportion of catastrophic costs (36% vs. 65%) than estimated in the meta-regression.*

**Author’s response: Your commentary is appreciated. The discussion was then adjusted to first comment on the main results (lines: 923-935). However, a difference of 68 percentage points in employment status at the start of treatment and at the interview was observed among study participants. It is important to note that if this is not taken into consideration when calculating indirect costs, as the primary indicator, this may result in an underestimation of income loss and, consequently, the indicator in scenarios such as Guatemala. This suggests that the actual burden of catastrophic costs of tuberculosis in the country is consistent with the sensitivity analyses, whose values are included in the uncertainty level of the referred study.**

*9. In the discussion, it is proposed that active case finding may reduce the catastrophic cost burden by reducing diagnostic delay. This may be true, but this statement is not corroborated by the findings of this study, which found no association in catastrophic costs with treatment delay.*

**Author’s response: Please note that the aforementioned excerpt addresses direct medical costs exclusively, not the catastrophic costs that were the subject of the association analysis. In any case, we expanded the discussion and clarified these differences (lines: 980-984).**

*10. In the discussion, it is said the poorest population is subject to the greatest catastrophic costs, but that is not the case in the main analysis, where catastrophic costs are lowest among the poorest quintile.*

**Author’s response: Your commentary is appreciated. We have restructured this segment of the discussion in order to elucidate the primary findings, the findings of the sensitivity analysis, and our interpretation of each (lines: 1001-1009).**

*11. The authors do propose that the human capital approach may be more appropriate for their context given the informal sector. It is therefore curious why an output approach was used in the first place, as the WHO suggests the output approach be avoided in settings with large informal economies. This needs to be carefully justified and explained.*

**Author’s response: Both measures reveal advantages and disadvantages. Each of these metrics focuses on a distinct aspect of indirect costs, yet neither offers a comprehensive assessment. Therefore, given the recommendation that both should be presented and interpreted in a comprehensive manner,(3) and the fact that the WHO will monitor the indicator estimated by the output approach for the country's targets, this outcome was identified as the primary one. We have made this more explicit in the text (lines: 389-392; 888-900) and have also emphasized that for both, we consider that adjustment for the current employment status is essential to avoid underestimations.**

*12. The underlying data should be made available during peer review.*

**Author’s response: We apologize for this. The data can be accessed via the link:** https://doi.org/10.6084/m9.figshare.31708996**, which will be made public if the manuscript is accepted for publication.**

***Opinion attached:***

*Thank you for the opportunity to review this manuscript. The co-authors should be commended for strong writing and for producing Guatemala’s first national TB patient cost survey. The manuscript addresses an important evidence gap in the region, and the analysis is timely and policy-relevant.*

*Below, I outline suggestions that may strengthen clarity, methodological transparency, and interpretive depth.*

**Author’s response: We acknowledge and sincerely appreciate the thorough evaluation and contributions made. A comprehensive revision of the manuscript was undertaken to incorporate the suggestions.**

*Abstract*

*Consider defining catastrophic costs directly in the abstract to improve accessibility for readers less familiar with TB cost-survey methodologies*

**Author’s response: The definition was included (lines: 32-34)**

*Introduction*

*Tuberculosis is written out in most instances; however, the accepted abbreviation TB could be used more consistently to improve readability.*

**Author’s response: We have modified the original text in accordance with the recommendations.**

*Third paragraph of the Introduction should be copy edited. You are missing a parenthesis after 48%. The phrase “actual situation” could be replaced with more precise scientific language. The last sentence of the paragraph repeats the word strategies and could be streamlined for clarity.*

**Author’s response: Thank you for your comment. The revision has been made (lines: 78; 81).**

*Methods*

*The authors should justify why the output approach was selected as the main method for estimating indirect costs. The WHO handbook recommends its use in predominantly formal economies, yet the study population shows a high level of informal employment. Additional explanation is needed to clarify why this approach is most appropriate in this setting.*

**Author’s response: This recommendation aligns with the guidelines outlined in the WHO handbook, published in 2017 [1]. However, as referenced in the manuscript, the analyses were conducted based on the update to this manual, published in 2025 [3]. As stated in the recent publication's key updates section, indirect costs are estimated through the application of two distinct methodologies: the output approach and the human capital approach [3]. The former is utilized in the primary estimation of catastrophic costs, while the latter is explored and discussed in the context of sensitivity analysis [3]. As previously stated, the rationale for this modification is that the two metrics are distinct and each one is limited in its ability to capture all indirect costs associated with tuberculosis [3]. Furthermore, if necessary, additional sensitivity analyses are recommended [3]. In this context, we employed additional analyses given the scenario of change in the employment status of participants.**

**A further noteworthy point relates to the initial justification for the recommendation to avoid the output approach, which was the potential for fluctuations in income in settings characterized by a large informal labor market. However, when income was calculated based on household characteristics, the values were close to self-reported income. This finding supports the reliability of the results and was incorporated into the manuscript (lines: 389-392; 888-900; 906-910).**

*I would suggest that the Measurements section should precede Variables, as the current order left several variable definitions unclear during initial reading. If the authors prefer, they could also create a box for the definition of variables.*

**Author’s response: In accordance with the recommendation, the subsection's positioning was altered (lines: 139-188), and the text was revised to ensure a more fluid reading.**

*The authos should provide clearer definitions for ‘ever hospitalized’. Does this mean during the episode of TB or throughout the person’s life?*

**Author’s response: The definition of the variable has been revised (lines: 229-230). It concerns the occurrence of hospitalizations during current treatment for tuberculosis.**

*“Social support” includes what types? Define Any social effect should state the specific items included.*

**Author’s response: The description of the variables was modified to emphasize the derivation process (lines: 198-200).**

*The section labeled Bias describes steps taken to reduce bias, not the sources of bias*

*themselves. Consider renaming it to Bias Handling, Mitigation of Bias, or a similar term.*

**Author’s response: The adjustment was performed as recommended (line: 232).**

*Quality control of data entry should be better explained.*

**Author’s response: The description has been expanded as recommended (lines: 243-246).**

*The pilot study is briefly mentioned but could be described more fully (e.g., sample used, changes made following the pilot).*

**Author’s response: The description has been expanded as recommended (lines: 239-242).**

*The study does not provide sufficient information about the selection of clusters. More*

*information is needed on how clusters were distributed across regions and facilities. The sample size calculation specifies 44 clusters, yet only 42 were used. Clarification is needed on why two clusters were not included and whether this affects representativeness.*

**Author’s response: The explanation of the sampling process was expanded to include details about its execution according to regional strata of tuberculosis case distribution in the country (lines: 341-342). Furthermore, we have provided additional clarifications regarding the rationale and methodology employed in addressing the loss of the two clusters (lines: 347-349; 444-447).**

*The description of extrapolation methods in the Statistical Methods section should be*

*expanded to ensure transparency.*

Author’s response: **Given that this analysis stage has been previously delineated in a series of publications, including earlier articles, our initial approach was to streamline the description and incorporate the pertinent reference. However, we have expanded the description, as recommended by the reviewer (lines: 350-356).**

*Chart 1 indicates rules for “Employed before onset of TB,” but the accompanying text suggests these rules apply to income before and during treatment. This requires clarification. Please provide a rationale for why missing income values were replaced with zero under the output approach, yet estimated using average wages or minimum wages under the human capital approach. It may introduce systematic bias.*

*In addition to missing values, the manuscript should clarify how zero income was handled analytically.*

**Author’s response: The distinction in their handling, as recommended by the WHO, is rooted in the conceptual differences in the metrics assessed by each approach. The output approach attempts to evaluate the actual loss of income in the labor market (i.e., the absence of employment results in a lack of income). Conversely, the human capital approach focuses on the potential value of the individual, considering their capacity to generate well-being and production, irrespective of their participation in the labor market [4]. In scenarios such as Guatemala, involving changes in employment status, there is a possibility of the introduction of systematic error if the employment situation at the time of the interview is not taken into account when estimating current income. However, to address this situation, sensitivity analyses were included, as discussed above (lines: 389-392; 888-895; 906-910). Concerning zeros, they were addressed as inconsistencies or not, depending on the employment situation, as the text further elaborates (lines: 366-372).**

*Results*

*The results are clearly presented; figures and tables are aligned with WHO reporting.*

*The eight development regions are currently listed alphabetically. Guatemala typically numbers its regions; arranging them in numeric or geographic order may improve interpretability.*

**Author’s response: The sequence of the regions has been modified and numbering has been incorporated, as recommended (lines: 209-210).**

*The reversal of the wealth gradient between main and sensitivity analyses is particularly important but is treated briefly.*

**Author’s response: We opted for a concise presentation of this result, ensuring it remained within the scope of the description, a choice that aligns with the objectives of this section. However, in the subsequent section (Discussion), the findings were subjected to a more extensive interpretation and discussion (lines: 1002-1010).**

*Some regions (e.g., Petén, North West) have very small denominators (1–5 participants), raising concerns about the stability of prevalence ratio estimates.*

**Author’s response: As previously stated, the sampling process was designed to account for the regional distribution of cases. However, it is important to note that the objective of the study, and consequently its sample size calculation, was to present an estimate of catastrophic costs for the country. Accordingly, the manuscript presents distributions of the variables of interest in order to characterize the population, and this information was reinforced in the methods section (line: 395).**

*The hierarchical regression framework is appropriate, but the conceptual model used to assign variables to levels is not sufficiently described. The linkage between the theoretical model in Methods and Tables 3 &amp; 4 should be strengthened.*

**Author’s response: As suggested, the definition of hierarchical models has been expanded in the manuscript (lines: 418-423).**

*The manuscript refers to “access” to coping strategies. While access to credit may be*

*constrained, the sale of assets is generally not an issue of access. The authors may wish to reconsider this terminology and provide a full definition of the coping strategies assessed.*

**Author’s response: The definition has been improved (lines: 200-202), and an additional consideration has been included in the discussion on the non-use of coping strategies (lines: 1005-1009; 1065-1067).**

*Citation 13 appears to be incomplete; I was unable to retrieve it with the information provided*

**Author’s response: The reference has been updated (lines: 1153-1156) and all others have been reviewed to ensure consistency (lines: 1110-1199).**

*Discussion*

*The Discussion synthesizes findings well and situates Guatemala within global patterns of catastrophic costs. Policy recommendations are aligned with WHO guidance.*

**Author’s response: We appreciate your comments.**

*The interpretation of the large discrepancies observed between the main and sensitivity analyses could be expanded.*

**Author’s response: The discussion was expanded, thereby reinforcing the methodological differences and specificities of the study population, as suggested (lines: 923-935).**

*Greater attention could be given to Guatemala’s health-system context, particularly its*

*extremely high out-of-pocket expenditure, fragmented and underfunded public services, and limited, uneven social assistance coverage. The manuscript could also elaborate on health financing structures in Guatemala, providing context for why TB-affected households face high direct medical and non-medical costs.*

**Author’s response: Potential explanations for the elevated medical expenditures have been incorporated to the manuscript (lines: 944-965).**

*Recommendations related to social protection could be more concrete (e.g., transport*

*subsidies, nutritional support, labor protections, integration with existing cash-transfer*

*schemes).*

**Author’s response: The additional recommendations have been incorporated into the manuscript as suggested (lines: 951=965; 981-984).**

*The authors should consider incorporating critiques of income-based approaches to estimating financial hardship. For example, the following article offers relevant conceptual framing: Timire et al., Clinical Infectious Diseases (2023), “Use of a Sustainable Livelihood Framework–Based Measure to Estimate Socioeconomic Impact of TB on Households.”*

**Author’s response: We appreciate the suggestion. The discussion on this aspect was further expanded in the manuscript to emphasize that the range of analyses employed, when considered in a complementary manner, offer a more comprehensive perspective on the implications of tuberculosis treatment (lines: 889-893).**

*While the suggestions to reduce medical costs through active case finding, streamlining service delivery, and VOT are important, the Discussion could additionally highlight broader social-protection measures capable of reducing both direct and indirect costs.*

**Author’s response: The recommendations have been revised in order to ensure greater comprehensiveness, as was recommended (lines: 944-965; 981-1000).**

References

1 Tuberculosis patient cost surveys: A handbook. Geneva: World Health Organization; 2017 (https:// iris.who.int/handle/10665/259701).
2 Consolidated guidance on tuberculosis data generation and use. Module 4. Surveys of costs faced by households affected by tuberculosis. Geneva: World Health Organization; 2025 (<https://iris.who.int/bitstream/handle/10665/381505/>).
3 Schisterman EF, Cole SR, Platt RW. Overadjustment bias and unnecessary adjustment in epidemiologic studies. Epidemiology. 2009 Jul 1;20(4):488-95.
4 Drummond MF, Sculpher MJ, Claxton K, Stoddart GL, Torrance GW. Methods for the economic evaluation of health care programmes. Oxford university press; 2015 Sep 25.
