# Supplementary material for: Burden of catastrophic costs, income inequalities and associated factors in Guatemala: First national tuberculosis costs survey, 2023
Source: PLoS One. 2026 Aug 17;21(8):e0351164. doi: 10.1371/journal.pone.0351164 (PMC13480651; doi:10.1371/journal.pone.0351164)
Supplement: Supplementary file 4 [file pone.0351164.s005.docx]

**Response to reviewers**

Dear Reviewers,

We would once again like to thank you for your contributions to the manuscript. We have attempted to address the suggestions made.

In the following section, we provide a comprehensive response to each of the comments.

**Major Comments**

1. **Methods**

- While the sampling framework (cluster design, ICC, regional stratification) is described well, participant selection within facilities remains unclear.

**Author’s response: As recommended, additional information has been included in the 'Study Size and sampling' section (previously titled 'Study Size'), lines: 238-240.**

- Please clarify how participants were recruited at the facility level and how representation across intensive vs. continuation phases was achieved.

**Author’s response: As recommended, additional information has been included in the 'Study Size and sampling' (previously titled 'Study Size') and the Discussion sections, lines: 238-240; 508-510.**

- The handling of missing and zero income values differs across approaches (e.g., replacement with zero vs. imputed wages), which may introduce systematic bias in the estimation of indirect costs. This warrants clearer justification and discussion of potential implications.

**Author’s response: An additional consideration regarding this topic was included in the study’s limitations (lies: 520-522).**

- The description of cost extrapolation across treatment phases would benefit from clearer explanation within the statistical methods.

**Author’s response: The manuscript provided a textual description of the statistical method for extrapolating costs beyond the current phase (lines: 254-257).**

- The variable “social support” is included in the analysis but not clearly defined. Please specify what constitutes social support. Similarly, ensure that all composite variables (e.g., social effects, coping strategies) are clearly and consistently defined.

**Author’s response: Additional definitions have been included in the methods section (lines:198-200).**

2. **Results**

- Consider including an additional table presenting disaggregated costs (direct medical, direct non-medical, indirect costs), which would improve interpretability and align with standard TB cost survey reporting.

**Author’s response: The table has been included as recommended (lines: 392-395). The authors initially decided not to include the table because this is an original article, which assumes that the data are unpublished. As noted, the table follows the WHO standard for reporting the results of cost studies and is included in the study report.**

3. **Discussion**

- The Discussion would benefit from a clearer structure, with the key findings from Guatemala presented and interpreted upfront, before moving to strengths and limitations.

**Author’s response: In line with best practices, the first paragraph of the discussion summarizes the study’s main findings (lines: 476-487) and followed by the strengths and limitations.**

.

- The manuscript engages with the updated WHO guidance recommending the output approach as the primary measure and makes a clear effort to present complementary analyses. However, this important methodological tension could be more explicitly foregrounded and analytically developed. In particular, the reversal of the wealth gradient between the main and sensitivity analyses is a central finding that warrants deeper interpretation, rather than primarily methodological explanation. The Discussion would benefit from more clearly articulating what this divergence implies for the validity of the primary measure in Guatemala’s context of high labor informality, and from taking a more explicit position on how WHO-recommended approaches perform in such settings. Strengthening this interpretation would enhance both the scientific contribution and the policy relevance of the manuscript.

**Author’s response: This topic was discussed in greater depth (lines: 604-609).**

- The Discussion could also be strengthened by engaging with critiques of income-based measures of financial hardship. For example, work using a sustainable livelihoods framework (e.g., Timire et al., 2023) may provide a useful starting point.

**Author’s response: We believe that this topic has been adequately covered by the additional references included.**

- Finally, increased engagement with the WHO guidance on social protection for TB-affected people and the Multisectoral Accountability Framework would further strengthen the policy framing around potential strategies to reduce catastrophic costs.

**Author’s response: The recommended reference has been included (lines: 547-549; 684-685).**

**Minor Comments**

4. **Abstract**

- The abstract could be strengthened by presenting results more quantitatively, particularly for associated factors, social effects, and coping strategies.

**Author’s response: The passage has been revised to include the data (lines 44-50).**

5. **Introduction**

- The statement that TB cost survey data have informed policy could be strengthened by referencing broader global examples. Fo example, see: <https://www.ncbi.nlm.nih.gov/pmc/articles/PMC6645445/>

**Author’s response: We believe that the references currently included, which include review articles, are sufficient to support this statement.**

6. **Consistency**

- Standardize references to the Ministry of Health (currently appears as “Ministry of Health,” “Guatemala Ministry of Health,” and “Ministry of Health and Social Assistance”).

**Author’s response: The manuscript has been revised.**

- Figure numbering and sequencing should be carefully checked, as there appear to be inconsistencies.

**Author’s response: The figure numbering has been revised.**

- The manuscript uses inconsistent numbers of decimal places across tables and text; these should be standardized to improve clarity and consistency.

**Author’s response: In accordance with best practices, decimal digits were standardized as follows: none for percentages, one for means, two for effect sizes and three for p-values.**

7. **Justification of exclusion criteria**

- The exclusion of individuals experiencing homelessness should be briefly justified, given their likely vulnerability to catastrophic costs.

**Author’s response: The justification has been included (lines: 130-131).**

8. **Redundancy and repetition**

- Survey dates are repeated multiple times across sections.

**Author’s response: The inclusion was requested by the reviewers.**

- The paragraph following Chart 1 is repetitive and could be condensed.

**Author’s response: The text was expanded upon the request of the reviewers.**

- The hierarchical frameworks across outcomes are very similar and could potentially be streamlined.

**Author’s response: The text was expanded upon the request of the reviewers.**

9. **Language and style**

- Additional copyediting is needed to address minor grammatical issues and inconsistencies.

**Author’s response: The text underwent further revision in order to address the requests.**

- Contractions in the Discussion should be removed to maintain a formal tone.

**Author’s response: The text underwent further revision in order to address the requests.**

- The final paragraph of the Conclusions differs stylistically and should be aligned with the rest of the manuscript.

**Author’s response: The text underwent further revision in order to address the requests (lines: 692-695).**
