# Supplementary material for: Burden of catastrophic costs, income inequalities and associated factors in Guatemala: First national tuberculosis costs survey, 2023
Source: PLoS One. 2026 Aug 17;21(8):e0351164. doi: 10.1371/journal.pone.0351164 (PMC13480651; doi:10.1371/journal.pone.0351164)
Supplement: Peer review history

# Peer review: Decision Letter 0

**DOI:** 10.1371/journal.pone.0351164.r001

**Authors:** Tom Wingfield

6 Jan 2026

Dear Dr. Reis-Santos,

Thank you for submitting your manuscript on TB patient costs in Guatemala to PLOS One. Both reviewers commended the importance, timeliness, and policy relevance of this work, particularly as the first national TB patient cost survey in the country. They also identified several key areas that require careful attention in revision.

Across both reviews, the most critical issues related to methodological transparency, internal consistency, and interpretation. In particular, please:

1. Clarify and justify methodological choices, especially the use of the output approach as the primary method for estimating indirect costs in a setting with a large informal economy, given WHO guidance. Expand the description of key equations, assumptions, handling of zero and missing income values, extrapolation methods, and the rationale for differences between the output and human capital approaches.

2. Strengthen reporting of the sampling design, including cluster selection, cluster size, ICC and design effect, regional distribution of clusters, and clarification of discrepancies between planned and achieved clusters. Provide precise study dates in the Methods and Results.

3. Improve clarity and consistency in definitions and structure, including clearer variable definitions (e.g., hospitalization, social support, social effects, coping strategies), reordering or restructuring sections where helpful, and clearer linkage between the conceptual framework and regression models. Please also comment on potential over-adjustment in regression analyses given outcome prevalence.

4. Address discrepancies between main and sensitivity analyses, particularly the reversal of the wealth gradient and unexpected findings such as higher catastrophic costs in richer quintiles. Ensure that the abstract reflects the main analysis rather than sensitivity analyses, and expand discussion of these differences.

5. Revise the Discussion for accuracy and alignment with results, avoiding claims not supported by the study (e.g., effects of active case finding or poorest quintiles bearing the highest burden), and more carefully situating Guatemala’s findings relative to global estimates. Additional contextualization of Guatemala’s health financing and social protection landscape would strengthen the policy relevance of your paper.

6. Attend to presentation and completeness, including careful proofreading, correction of minor errors and missing citations, clearer figure/table explanations, consideration of a map of study facilities, and ensuring data availability in line with PLOS One requirements and reviewer comments.

Addressing these points should substantially improve the manuscript for our broad PLOS One readership. We look forward to receiving a revised version that responds thoroughly to the reviewers’ comments and thank you again for your hard work.

Kind regards,

==============================

Please submit your revised manuscript by Feb 20 2026 11:59PM. If you will need more time than this to complete your revisions, please reply to this message or contact the journal office at plosone@plos.org. When you're ready to submit your revision, log on to https://www.editorialmanager.com/pone/ and select the 'Submissions Needing Revision' folder to locate your manuscript file.

- A letter that responds to each point raised by the academic editor and reviewer(s). You should upload this letter as a separate file labeled 'Response to Reviewers'.
- A marked-up copy of your manuscript that highlights changes made to the original version. You should upload this as a separate file labeled 'Revised Manuscript with Track Changes'.
- An unmarked version of your revised paper without tracked changes. You should upload this as a separate file labeled 'Manuscript'.

If you would like to make changes to your financial disclosure, please include your updated statement in your cover letter. Guidelines for resubmitting your figure files are available below the reviewer comments at the end of this letter.

If applicable, we recommend that you deposit your laboratory protocols in protocols.io to enhance the reproducibility of your results. Protocols.io assigns your protocol its own identifier (DOI) so that it can be cited independently in the future. For instructions see: https://journals.plos.org/plosone/s/submission-guidelines#loc-laboratory-protocols. Additionally, PLOS ONE offers an option for publishing peer-reviewed Lab Protocol articles, which describe protocols hosted on protocols.io. Read more information on sharing protocols at https://plos.org/protocols?utm_medium=editorial-email&utm_source=authorletters&utm_campaign=protocols.

We look forward to receiving your revised manuscript.

Kind regards,

Tom E. Wingfield

Academic Editor

PLOS One

Journal Requirements:

When submitting your revision, we need you to address these additional requirements.

1. Please ensure that your manuscript meets PLOS ONE's style requirements, including those for file naming. The PLOS ONE style templates can be found at

https://journals.plos.org/plosone/s/file?id=wjVg/PLOSOne_formatting_sample_main_body.pdf and

https://journals.plos.org/plosone/s/file?id=ba62/PLOSOne_formatting_sample_title_authors_affiliations.pdf.

2. Please include a complete copy of PLOS’ questionnaire on inclusivity in global research in your revised manuscript. Our policy for research in this area aims to improve transparency in the reporting of research performed outside of researchers’ own country or community. The policy applies to researchers who have travelled to a different country to conduct research, research with Indigenous populations or their lands, and research on cultural artefacts. The questionnaire can also be requested at the journal’s discretion for any other submissions, even if these conditions are not met. Please find more information on the policy and a link to download a blank copy of the questionnaire here: https://journals.plos.org/plosone/s/best-practices-in-research-reporting. Please upload a completed version of your questionnaire as Supporting Information when you resubmit your manuscript.

3. Thank you for stating in your Funding Statement:

“The study received financial support from the Global Fund (https://www.theglobalfund.org/en/). The funding source had no involvement in the design, execution, analysis, interpretation of the data, or the decision to publish the manuscript.”

Please provide an amended statement that declares *all* the funding or sources of support (whether external or internal to your organization) received during this study, as detailed online in our guide for authors at http://journals.plos.org/plosone/s/submit-now.  Please also include the statement “There was no additional external funding received for this study.” in your updated Funding Statement.

Please include your amended Funding Statement within your cover letter. We will change the online submission form on your behalf.

4. Please note that your Data Availability Statement is currently missing [the repository name and/or the DOI/accession number of each dataset OR a direct link to access each database]. If your manuscript is accepted for publication, you will be asked to provide these details on a very short timeline. We therefore suggest that you provide this information now, though we will not hold up the peer review process if you are unable.

5. Please be informed that funding information should not appear in the Acknowledgments section or other areas of your manuscript. We will only publish funding information present in the Funding Statement section of the online submission form. Please remove any funding-related text from the manuscript.

6. If the reviewer comments include a recommendation to cite specific previously published works, please review and evaluate these publications to determine whether they are relevant and should be cited. There is no requirement to cite these works unless the editor has indicated otherwise.

[Note: HTML markup is below. Please do not edit.]

Reviewers' comments:

Reviewer's Responses to Questions

**Comments to the Author**

1. Is the manuscript technically sound, and do the data support the conclusions?

Reviewer #1: Yes

Reviewer #2: Partly

**********

2. Has the statistical analysis been performed appropriately and rigorously?

Reviewer #1: Yes

Reviewer #2: No

**********

3. Have the authors made all data underlying the findings in their manuscript fully available?

The PLOS Data policy

Reviewer #1: No

Reviewer #2: No

**********

4. Is the manuscript presented in an intelligible fashion and written in standard English?

Reviewer #1: Yes

Reviewer #2: No

**********

Reviewer #1: The authors have written that they will make their data available once the manuscript has been approved, but at the present moment, the manuscript does not align with PLOS One's Data Policy. The editor may need to request that the authors either provide full access to the dataset prior to further review or formally justify an exemption under PLOS ONE’s data-availability guidelines before the manuscript can proceed in the review process.

Reviewer #2: The authors present a study of patient costs among people with TB in Guatemala in 2023. The authors found 36% of participants experienced catastrophic costs, using the output approach. Catastrophic costs were more common among the youngest age groups. This is an important question and I commend the authors for seeking to contribute to a nascent literature in the Americas. There are likely important insights that can be gleaned from this work. I have several comments for improvement.

1. Please report the cluster size and ICC used to calculate the design effect.

2. Please provide precise dates of the study when you mention it. 2023 is fine for the title, but precise dates should be provided in the methods and results (I recognize it is provided once).

3. The abstract appears to report a sensitivity analysis by income quintile, rather than the main analysis. The numbers appear highly unlikely.

4. PLoS One is a general journal. It would be helpful to expand upon the methods and equations used to estimate key outcomes (e.g., equations used for the output approach, human capital approach). This is missing and would help make clear to readers why some unexpected findings (e.g., catastrophic costs largest in the richest quintile) occur.

5. I do not believe PLoS One provides copyediting. The manuscript should be proofread carefully and corrected for grammar.

6. Readers may find it helpful to have a map of the included health facilities in Guatemala.

7. The regression analyses may be over-adjusted based on the prevalence of the outcome, particularly for catastrophic costs. This should be commented on.

8. In the discussion, it is stated that the findings of this analysis are congruent with a global meta-regression. However, this is not the case for the study's main analysis. Indeed, the majority of studies informing the global meta-regression used the output approach, and so it appears Guatemala may have a much lower proportion of catastrophic costs (36% vs. 65%) than estimated in the meta-regression.

9. In the discussion, it is proposed that active case finding may reduce the catastrophic cost burden by reducing diagnostic delay. This may be true, but this statement is not corroborated by the findings of this study, which found no association in catastrophic costs with treatment delay.

10. In the discussion, it is said the poorest population is subject to the greatest catastrophic costs, but that is not the case in the main analysis, where catastrophic costs are lowest among the poorest quintile.

11. The authors do propose that the human capital approach may be more appropriate for their context given the informal sector. It is therefore curious why an output approach was used in the first place, as the WHO suggests the output approach be avoided in settings with large informal economies. This needs to be carefully justified and explained.

12. The underlying data should be made available during peer review.

**********

what does this mean?). If published, this will include your full peer review and any attached files.

If you choose “no”, your identity will remain anonymous but your review may still be made public.

**Do you want your identity to be public for this peer review?**  For information about this choice, including consent withdrawal, please see our Privacy Policy

Reviewer #1: No

Reviewer #2: No

**********

[NOTE: If reviewer comments were submitted as an attachment file, they will be attached to this email and accessible via the submission site. Please log into your account, locate the manuscript record, and check for the action link "View Attachments". If this link does not appear, there are no attachment files.]

To ensure your figures meet our technical requirements, please review our figure guidelines: https://journals.plos.org/plosone/s/figures

You may also use PLOS’s free figure tool, NAAS, to help you prepare publication quality figures: https://journals.plos.org/plosone/s/figures#loc-tools-for-figure-preparation.

NAAS will assess whether your figures meet our technical requirements by comparing each figure against our figure specifications.

Attachment Submitted filename: Guatemala PCS.docx

# Author comment: Author response to Decision Letter 1

**DOI:** 10.1371/journal.pone.0351164.r002

31 Mar 2026

Response to reviewers

Dear Editor and Reviewers,

We are grateful for all the comments and contributions that have been provided, which have undoubtedly contributed to the enhancement of our manuscript.

We have attempted to address all the suggestions made.

In the following section, we provide a comprehensive response to each of the aforementioned comments.

Editor’s comments:

Across both reviews, the most critical issues related to methodological transparency, internal consistency, and interpretation. In particular, please:

1. Clarify and justify methodological choices, especially the use of the output approach as the primary method for estimating indirect costs in a setting with a large informal economy, given WHO guidance. Expand the description of key equations, assumptions, handling of zero and missing income values, extrapolation methods, and the rationale for differences between the output and human capital approaches.

Author’s response: With regard to indicators, the WHO guide published in 2017 [1] actually advised against the use of the output approach for scenarios with a high proportion of informal labor market. However, the most recent publication in 2025 [2] has introduced a significant alteration to this approach. The new guideline advocates for analyses to encompass both approaches, with output designated as the primary indicator and human capital used for sensitivity analysis [2]. This shift in recommendation is attributable to the findings highlighting diverse and complementary dimensions of the object of study. Furthermore, additional analyses were incorporated to address the change in employment status that occurred among the study participants. This information was elucidated in the text (lines: 389-392; 888-900).

Subsequent modifications were made to the text of the manuscript in order to ensure that a comprehensive description of the key equations (lines: 158-173; 352-353), assumptions (lines: 198-201; 336-337; 401-405; 418-423), handling of zero and missing income values (lines: 357-373), and extrapolation methods (lines: 350-356) was provided.

2. Strengthen reporting of the sampling design, including cluster selection, cluster size, ICC and design effect, regional distribution of clusters, and clarification of discrepancies between planned and achieved clusters. Provide precise study dates in the Methods and Results.

Author’s response: Further information has been provided on sample design, sampling process, and losses (lines: 336-337; 341-342; 346-349; 444-447). In addition, the precise dates of data collection were replicated in the different sections (lines: 111; 443).

3. Improve clarity and consistency in definitions and structure, including clearer variable definitions (e.g., hospitalization, social support, social effects, coping strategies), reordering or restructuring sections where helpful, and clearer linkage between the conceptual framework and regression models. Please also comment on potential over-adjustment in regression analyses given outcome prevalence.

Author’s response: The text has been restructured in accordance with the recommendations (lines: 139-231). The definitions of variables have undergone a revision (lines: 198-231). The rationale supporting the theoretical model and the defined hierarchical levels has also been incorporated (lines: 401-405), in addition to the discussion on potential overfitting (lines: 418-423).

In relation to the final topic, it is important to emphasize that the regression models implemented in the study were structured to minimize the probability of overadjustment. In spite of the cross-sectional nature of the study, it can be asserted that the independent variables included in the model precede catastrophic costs, which is an outcome of treatment [3]. Moreover, the hierarchical entry of independent variables into the model avoided adjustment by intermediate variables [3].

4. Address discrepancies between main and sensitivity analyses, particularly the reversal of the wealth gradient and unexpected findings such as higher catastrophic costs in richer quintiles. Ensure that the abstract reflects the main analysis rather than sensitivity analyses, and expand discussion of these differences.

Author’s response: The discussion related to this aspect has been improved (lines: 1002-1010), and all necessary information for correct interpretation has been included in the abstract (lines: 31-43).

5. Revise the Discussion for accuracy and alignment with results, avoiding claims not supported by the study (e.g., effects of active case finding or poorest quintiles bearing the highest burden), and more carefully situating Guatemala’s findings relative to global estimates. Additional contextualization of Guatemala’s health financing and social protection landscape would strengthen the policy relevance of your paper.

Author’s response: The discussion section has undergone extensive revision to ensure that all information presented is supported. The discussion of the findings (lines: 1002-1010; 923-935) and the contextualization of the estimates has been advanced (lines: 944-965).

6. Attend to presentation and completeness, including careful proofreading, correction of minor errors and missing citations, clearer figure/table explanations, consideration of a map of study facilities, and ensuring data availability in line with PLOS One requirements and reviewer comments.

Author’s response: The text, references and illustrations have been revised. The map has been incorporated in accordance with the recommendation. The data can be accessed via the link: https://doi.org/10.6084/m9.figshare.31708996

Reviewers' comments:

1. Is the manuscript technically sound, and do the data support the conclusions?

Reviewer #1: Yes

Reviewer #2: Partly

Author’s response: The manuscript has undergone a comprehensive revision to ensure that all information supporting the conclusions is explicitly described.

2. Has the statistical analysis been performed appropriately and rigorously?

Reviewer #1: Yes

Reviewer #2: No

Author’s response: The description of statistical methods was improved to ensure that all strategies adopted were detailed, as recommended by the reviewers.

3. Have the authors made all data underlying the findings in their manuscript fully available?

Reviewer #1: No

Reviewer #2: No

Author’s response: The data can be accessed via the link: https://doi.org/10.6084/m9.figshare.31708996

4. Is the manuscript presented in an intelligible fashion and written in standard English?

Reviewer #1: Yes

Reviewer #2: No

Author’s response: The manuscript has undergone extensive revision to ensure compliance with academic standards.

5. Review Comments to the Author

Reviewer #1: The authors have written that they will make their data available once the manuscript has been approved, but at the present moment, the manuscript does not align with PLOS One's Data Policy. The editor may need to request that the authors either provide full access to the dataset prior to further review or formally justify an exemption under PLOS ONE’s data-availability guidelines before the manuscript can proceed in the review process.

Author’s response: The data can be accessed via the link: https://doi.org/10.6084/m9.figshare.31708996

Reviewer #2: The authors present a study of patient costs among people with TB in Guatemala in 2023. The authors found 36% of participants experienced catastrophic costs, using the output approach. Catastrophic costs were more common among the youngest age groups. This is an important question and I commend the authors for seeking to contribute to a nascent literature in the Americas. There are likely important insights that can be gleaned from this work. I have several comments for improvement.

Author’s response: Thank you for your careful review and comments. We really appreciate the contributions they made to the improvement of the manuscript. We have attempted to address all the suggestions to the best of our ability.

1. Please report the cluster size and ICC used to calculate the design effect.

Author’s response: The passage was rewritten and the information was added to improve understanding (lines: 336-337).

2. Please provide precise dates of the study when you mention it. 2023 is fine for the title, but precise dates should be provided in the methods and results (I recognize it is provided once).

Author’s response: The precise dates on which the data were collected for the study were incorporated into both the design subsection (line: 111) and the presentation of results (line: 443). However, in the discussion section, the year (2023) was retained, given that the methods employed in the study enable the inference of results for that year.1

3. The abstract appears to report a sensitivity analysis by income quintile, rather than the main analysis. The numbers appear highly unlikely.

Author’s response: Indeed, the results presented are from the sensitivity analysis, and the methods do not provide adequate support for their interpretation. The text has been revised (lines: 32-43).

4. PLoS One is a general journal. It would be helpful to expand upon the methods and equations used to estimate key outcomes (e.g., equations used for the output approach, human capital approach). This is missing and would help make clear to readers why some unexpected findings (e.g., catastrophic costs largest in the richest quintile) occur.

Author’s response: The method for calculating the indicators had been defined in the variables’ subsection (lines: 153-168; 175-182). However, following the reviewer's request, the statistical methods were revisited and the information re-entered using figure with the equations [lines: 170 (Figure 1); 350-353].

5. I do not believe PLoS One provides copyediting. The manuscript should be proofread carefully and corrected for grammar.

Author’s response: We appreciate your comment. The manuscript has undergone extensive revision.

6. Readers may find it helpful to have a map of the included health facilities in Guatemala.

Author’s response: The map has been included and is presented as the new Figure 2 (line: 449).

7. The regression analyses may be over-adjusted based on the prevalence of the outcome, particularly for catastrophic costs. This should be commented on.

Author’s response: As recommended, a comment on this topic has been included in the methods (lines: 418-423; 893-900). The regression models implemented in the study were structured to minimize the probability of overadjustment. In spite of the cross-sectional nature of the study, it can be asserted that the independent variables included in the model precede catastrophic costs, which is an outcome of treatment.2 Moreover, the hierarchical entry of independent variables into the model avoided adjustment by intermediate variables.2

8. In the discussion, it is stated that the findings of this analysis are congruent with a global meta-regression. However, this is not the case for the study's main analysis. Indeed, the majority of studies informing the global meta-regression used the output approach, and so it appears Guatemala may have a much lower proportion of catastrophic costs (36% vs. 65%) than estimated in the meta-regression.

Author’s response: Your commentary is appreciated. The discussion was then adjusted to first comment on the main results (lines: 923-935). However, a difference of 68 percentage points in employment status at the start of treatment and at the interview was observed among study participants. It is important to note that if this is not taken into consideration when calculating indirect costs, as the primary indicator, this may result in an underestimation of income loss and, consequently, the indicator in scenarios such as Guatemala. This suggests that the actual burden of catastrophic costs of tuberculosis in the country is consistent with the sensitivity analyses, whose values are included in the uncertainty level of the referred study.

9. In the discussion, it is proposed that active case finding may reduce the catastrophic cost burden by reducing diagnostic delay. This may be true, but this statement is not corroborated by the findings of this study, which found no association in catastrophic costs with treatment delay.

Author’s response: Please note that the aforementioned excerpt addresses direct medical costs exclusively, not the catastrophic costs that were the subject of the association analysis. In any case, we expanded the discussion and clarified these differences (lines: 980-984).

10. In the discussion, it is said the poorest population is subject to the greatest catastrophic costs, but that is not the case in the main analysis, where catastrophic costs are lowest among the poorest quintile.

Author’s response: Your commentary is appreciated. We have restructured this segment of the discussion in order to elucidate the primary findings, the findings of the sensitivity analysis, and our interpretation of each (lines: 1001-1009).

11. The authors do propose that the human capital approach may be more appropriate for their context given the informal sector. It is therefore curious why an output approach was used in the first place, as the WHO suggests the output approach be avoided in settings with large informal economies. This needs to be carefully justified and explained.

Author’s response: Both measures reveal advantages and disadvantages. Each of these metrics focuses on a distinct aspect of indirect costs, yet neither offers a comprehensive assessment. Therefore, given the recommendation that both should be presented and interpreted in a comprehensive manner,(3) and the fact that the WHO will monitor the indicator estimated by the output approach for the country's targets, this outcome was identified as the primary one. We have made this more explicit in the text (lines: 389-392; 888-900) and have also emphasized that for both, we consider that adjustment for the current employment status is essential to avoid underestimations.

12. The underlying data should be made available during peer review.

Author’s response: We apologize for this. The data can be accessed via the link: https://doi.org/10.6084/m9.figshare.31708996, which will be made public if the manuscript is accepted for publication.

Opinion attached:

Thank you for the opportunity to review this manuscript. The co-authors should be commended for strong writing and for producing Guatemala’s first national TB patient cost survey. The manuscript addresses an important evidence gap in the region, and the analysis is timely and policy-relevant.

Below, I outline suggestions that may strengthen clarity, methodological transparency, and interpretive depth.

Author’s response: We acknowledge and sincerely appreciate the thorough evaluation and contributions made. A comprehensive revision of the manuscript was undertaken to incorporate the suggestions.

Abstract

Consider defining catastrophic costs directly in the abstract to improve accessibility for readers less familiar with TB cost-survey methodologies

Author’s response: The definition was included (lines: 32-34)

Introduction

Tuberculosis is written out in most instances; however, the accepted abbreviation TB could be used more consistently to improve readability.

Author’s response: We have modified the original text in accordance with the recommendations.

Third paragraph of the Introduction should be copy edited. You are missing a parenthesis after 48%. The phrase “actual situation” could be replaced with more precise scientific language. The last sentence of the paragraph repeats the word strategies and could be streamlined for clarity.

Author’s response: Thank you for your comment. The revision has been made (lines: 78; 81).

Methods

The authors should justify why the output approach was selected as the main method for estimating indirect costs. The WHO handbook recommends its use in predominantly formal economies, yet the study population shows a high level of informal employment. Additional explanation is needed to clarify why this approach is most appropriate in this setting.

Author’s response: This recommendation aligns with the guidelines outlined in the WHO handbook, p

Attachment Submitted filename: PONE-D-25-57446_Response to reviewers.docx

# Peer review: Decision Letter 1

**DOI:** 10.1371/journal.pone.0351164.r003

**Authors:** Tom Wingfield

8 May 2026

Dear Dr. Reis-Santos,

Thank you for submitting your manuscript to PLOS ONE. After careful consideration, we feel that it has merit but does not fully meet PLOS ONE’s publication criteria as it currently stands. Therefore, we invite you to submit a revised version of the manuscript that addresses the points raised during the review process.

Please submit your revised manuscript by Jun 22 2026 11:59PM. If you will need more time than this to complete your revisions, please reply to this message or contact the journal office at plosone@plos.org. When you're ready to submit your revision, log on to https://www.editorialmanager.com/pone/ and select the 'Submissions Needing Revision' folder to locate your manuscript file.

- A letter that responds to each point raised by the academic editor and reviewer(s). You should upload this letter as a separate file labeled 'Response to Reviewers'.
- A marked-up copy of your manuscript that highlights changes made to the original version. You should upload this as a separate file labeled 'Revised Manuscript with Track Changes'.
- An unmarked version of your revised paper without tracked changes. You should upload this as a separate file labeled 'Manuscript'.

If you would like to make changes to your financial disclosure, please include your updated statement in your cover letter. Guidelines for resubmitting your figure files are available below the reviewer comments at the end of this letter.

If applicable, we recommend that you deposit your laboratory protocols in protocols.io to enhance the reproducibility of your results. Protocols.io assigns your protocol its own identifier (DOI) so that it can be cited independently in the future. For instructions see: https://journals.plos.org/plosone/s/submission-guidelines#loc-laboratory-protocols. Additionally, PLOS ONE offers an option for publishing peer-reviewed Lab Protocol articles, which describe protocols hosted on protocols.io. Read more information on sharing protocols at https://plos.org/protocols?utm_medium=editorial-email&utm_source=authorletters&utm_campaign=protocols.

As the corresponding author, your ORCID iD is verified in the submission system and will appear in the published article. PLOS supports the use of ORCID, and we encourage all coauthors to register for an ORCID iD and use it as well. Please encourage your coauthors to verify their ORCID iD within the submission system before final acceptance, as unverified ORCID iDs will not appear in the published article. *Only*  the individual author can complete the verification step; PLOS staff *cannot*  verify ORCID iDs on behalf of authors.

We look forward to receiving your revised manuscript.

Kind regards,

Tom E. Wingfield

Academic Editor

PLOS One

Journal Requirements:

If the reviewer comments include a recommendation to cite specific previously published works, please review and evaluate these publications to determine whether they are relevant and should be cited. There is no requirement to cite these works unless the editor has indicated otherwise.

Please review your reference list to ensure that it is complete and correct. If you have cited papers that have been retracted, please include the rationale for doing so in the manuscript text, or remove these references and replace them with relevant current references. Any changes to the reference list should be mentioned in the rebuttal letter that accompanies your revised manuscript. If you need to cite a retracted article, indicate the article’s retracted status in the References list and also include a citation and full reference for the retraction notice.

Additional Editor Comments:

Dear Prof Reis-Santos and team,

Many thanks for your careful updates and improvements to the manuscript, which both I and the reviewers appreciate.

As you will note, there are still some minor elements that Reviewer 2 would like to see completed prior to publication.

I would be grateful if you could address these comments including adding a table of disaggregated medical costs, non-medical costs, and lost income, which aligns with many related publications on TB patient cost surveys and provides granular data with which to compare across other settings.

I will be glad to accept the updated version of the manuscript and please note that I would not be sending the revised version that you send back to me out for further reviewer comment.

Thank you for your time and efforts and we look forward to the updated version.

All the best,

Tom

[Note: HTML markup is below. Please do not edit.]

Reviewers' comments:

Reviewer's Responses to Questions

**Comments to the Author**

Reviewer #1: (No Response)

Reviewer #2: All comments have been addressed

**********

2. Is the manuscript technically sound, and do the data support the conclusions?

Reviewer #1: Yes

Reviewer #2: Yes

**********

3. Has the statistical analysis been performed appropriately and rigorously?

Reviewer #1: Yes

Reviewer #2: Yes

**********

4. Have the authors made all data underlying the findings in their manuscript fully available?

The PLOS Data policy

Reviewer #1: Yes

Reviewer #2: Yes

**********

5. Is the manuscript presented in an intelligible fashion and written in standard English?

Reviewer #1: Yes

Reviewer #2: Yes

**********

Reviewer #1: (No Response)

Reviewer #2: (No Response)

**********

what does this mean?). If published, this will include your full peer review and any attached files.

If you choose “no”, your identity will remain anonymous but your review may still be made public.

**Do you want your identity to be public for this peer review?**  For information about this choice, including consent withdrawal, please see our Privacy Policy

Reviewer #1: No

Reviewer #2: No

**********

[NOTE: If reviewer comments were submitted as an attachment file, they will be attached to this email and accessible via the submission site. Please log into your account, locate the manuscript record, and check for the action link "View Attachments". If this link does not appear, there are no attachment files.]

To ensure your figures meet our technical requirements, please review our figure guidelines: https://journals.plos.org/plosone/s/figures

You may also use PLOS’s free figure tool, NAAS, to help you prepare publication quality figures: https://journals.plos.org/plosone/s/figures#loc-tools-for-figure-preparation.

NAAS will assess whether your figures meet our technical requirements by comparing each figure against our figure specifications.

Attachment Submitted filename: Guatemala PCS_2.docx

# Author comment: Author response to Decision Letter 2

**DOI:** 10.1371/journal.pone.0351164.r004

21 May 2026

Dear Reviewers,

We would once again like to thank you for your contributions to the manuscript. We have attempted to address the suggestions made.

In the following section, we provide a comprehensive response to each of the comments.

Major Comments

1. Methods

• While the sampling framework (cluster design, ICC, regional stratification) is described well, participant selection within facilities remains unclear.

Author’s response: As recommended, additional information has been included in the 'Study Size and sampling' section (previously titled 'Study Size'), lines: 238-240.

• Please clarify how participants were recruited at the facility level and how representation across intensive vs. continuation phases was achieved.

Author’s response: As recommended, additional information has been included in the 'Study Size and sampling' (previously titled 'Study Size') and the Discussion sections, lines: 238-240; 508-510.

• The handling of missing and zero income values differs across approaches (e.g., replacement with zero vs. imputed wages), which may introduce systematic bias in the estimation of indirect costs. This warrants clearer justification and discussion of potential implications.

Author’s response: An additional consideration regarding this topic was included in the study’s limitations (lies: 520-522).

• The description of cost extrapolation across treatment phases would benefit from clearer explanation within the statistical methods.

Author’s response: The manuscript provided a textual description of the statistical method for extrapolating costs beyond the current phase (lines: 254-257).

• The variable “social support” is included in the analysis but not clearly defined. Please specify what constitutes social support. Similarly, ensure that all composite variables (e.g., social effects, coping strategies) are clearly and consistently defined.

Author’s response: Additional definitions have been included in the methods section (lines:198-200).

2. Results

• Consider including an additional table presenting disaggregated costs (direct medical, direct non-medical, indirect costs), which would improve interpretability and align with standard TB cost survey reporting.

Author’s response: The table has been included as recommended (lines: 392-395). The authors initially decided not to include the table because this is an original article, which assumes that the data are unpublished. As noted, the table follows the WHO standard for reporting the results of cost studies and is included in the study report.

3. Discussion

• The Discussion would benefit from a clearer structure, with the key findings from Guatemala presented and interpreted upfront, before moving to strengths and limitations.

Author’s response: In line with best practices, the first paragraph of the discussion summarizes the study’s main findings (lines: 476-487) and followed by the strengths and limitations.

• The manuscript engages with the updated WHO guidance recommending the output approach as the primary measure and makes a clear effort to present complementary analyses. However, this important methodological tension could be more explicitly foregrounded and analytically developed. In particular, the reversal of the wealth gradient between the main and sensitivity analyses is a central finding that warrants deeper interpretation, rather than primarily methodological explanation. The Discussion would benefit from more clearly articulating what this divergence implies for the validity of the primary measure in Guatemala’s context of high labor informality, and from taking a more explicit position on how WHO-recommended approaches perform in such settings. Strengthening this interpretation would enhance both the scientific contribution and the policy relevance of the manuscript.

Author’s response: This topic was discussed in greater depth (lines: 604-609).

• The Discussion could also be strengthened by engaging with critiques of income-based measures of financial hardship. For example, work using a sustainable livelihoods framework (e.g., Timire et al., 2023) may provide a useful starting point.

Author’s response: We believe that this topic has been adequately covered by the additional references included.

• Finally, increased engagement with the WHO guidance on social protection for TB-affected people and the Multisectoral Accountability Framework would further strengthen the policy framing around potential strategies to reduce catastrophic costs.

Author’s response: The recommended reference has been included (lines: 547-549; 684-685).

Minor Comments

4. Abstract

• The abstract could be strengthened by presenting results more quantitatively, particularly for associated factors, social effects, and coping strategies.

Author’s response: The passage has been revised to include the data (lines 44-50).

5. Introduction

• The statement that TB cost survey data have informed policy could be strengthened by referencing broader global examples. Fo example, see: https://www.ncbi.nlm.nih.gov/pmc/articles/PMC6645445/

Author’s response: We believe that the references currently included, which include review articles, are sufficient to support this statement.

6. Consistency

• Standardize references to the Ministry of Health (currently appears as “Ministry of Health,” “Guatemala Ministry of Health,” and “Ministry of Health and Social Assistance”).

Author’s response: The manuscript has been revised.

• Figure numbering and sequencing should be carefully checked, as there appear to be inconsistencies.

Author’s response: The figure numbering has been revised.

• The manuscript uses inconsistent numbers of decimal places across tables and text; these should be standardized to improve clarity and consistency.

Author’s response: In accordance with best practices, decimal digits were standardized as follows: none for percentages, one for means, two for effect sizes and three for p-values.

7. Justification of exclusion criteria

• The exclusion of individuals experiencing homelessness should be briefly justified, given their likely vulnerability to catastrophic costs.

Author’s response: The justification has been included (lines: 130-131).

8. Redundancy and repetition

• Survey dates are repeated multiple times across sections.

Author’s response: The inclusion was requested by the reviewers.

• The paragraph following Chart 1 is repetitive and could be condensed.

Author’s response: The text was expanded upon the request of the reviewers.

• The hierarchical frameworks across outcomes are very similar and could potentially be streamlined.

Author’s response: The text was expanded upon the request of the reviewers.

9. Language and style

• Additional copyediting is needed to address minor grammatical issues and inconsistencies.

Author’s response: The text underwent further revision in order to address the requests.

• Contractions in the Discussion should be removed to maintain a formal tone.

Author’s response: The text underwent further revision in order to address the requests.

• The final paragraph of the Conclusions differs stylistically and should be aligned with the rest of the manuscript.

Author’s response: The text underwent further revision in order to address the requests (lines: 692-695).

Attachment Submitted filename: Response to reviewers_2.docx

# Editor report: Decision Letter 2

**DOI:** 10.1371/journal.pone.0351164.r005

**Authors:** Tom Wingfield

24 May 2026

Burden of catastrophic costs, income inequalities and associated factors in Guatemala: first national tuberculosis costs survey, 2023

PONE-D-25-57446R2

Dear Dr. Reis-Santos,

We’re pleased to inform you that your manuscript has been judged scientifically suitable for publication and will be formally accepted for publication once it meets all outstanding technical requirements.

Within one week, you’ll receive an e-mail detailing the required amendments. When these have been addressed, you’ll receive a formal acceptance letter and your manuscript will be scheduled for publication.

An invoice will be generated when your article is formally accepted. Please note, if your institution has a publishing partnership with PLOS and your article meets the relevant criteria, all or part of your publication costs will be covered. Please make sure your user information is up-to-date by logging into Editorial Manager at Editorial Manager® and clicking the ‘Update My Information' link at the top of the page. For questions related to billing, please contact billing support.

If your institution or institutions have a press office, please notify them about your upcoming paper to help maximize its impact. If they’ll be preparing press materials, please inform our press team as soon as possible -- no later than 48 hours after receiving the formal acceptance. Your manuscript will remain under strict press embargo until 2 pm Eastern Time on the date of publication. For more information, please contact onepress@plos.org.

Kind regards,

Tom E. Wingfield

Academic Editor

PLOS One

Additional Editor Comments (optional):

Dear Prof Reis-Santos and team,

Thank you for your revision of the manuscript.

I have recommended your article for publication in PLOS One.

Many congratulations to you and the team and good luck for your future endeavours.

Tom Wingfield

Reviewers' comments:

# Editor report: Acceptance letter

**DOI:** 10.1371/journal.pone.0351164.r006

**Authors:** Tom Wingfield

PONE-D-25-57446R2

PLOS One

Dear Dr. Reis-Santos,

I'm pleased to inform you that your manuscript has been deemed suitable for publication in PLOS One. Congratulations! Your manuscript is now being handed over to our production team.

At this stage, our production department will prepare your paper for publication. This includes ensuring the following:

* All references, tables, and figures are properly cited

* All relevant supporting information is included in the manuscript submission,

* There are no issues that prevent the paper from being properly typeset

You will receive further instructions from the production team, including instructions on how to review your proof when it is ready. Please keep in mind that we are working through a large volume of accepted articles, so please give us a few days to review your paper and let you know the next and final steps.

Lastly, if your institution or institutions have a press office, please let them know about your upcoming paper now to help maximize its impact. If they'll be preparing press materials, please inform our press team within the next 48 hours. Your manuscript will remain under strict press embargo until 2 pm Eastern Time on the date of publication. For more information, please contact onepress@plos.org.

You will receive an invoice from PLOS for your publication fee after your manuscript has reached the completed accept phase. If you receive an email requesting payment before acceptance or for any other service, this may be a phishing scheme. Learn how to identify phishing emails and protect your accounts at https://explore.plos.org/phishing.

If we can help with anything else, please email us at customercare@plos.org.

Thank you for submitting your work to PLOS ONE and supporting open access.

Kind regards,

PLOS ONE Editorial Office Staff

on behalf of

Dr. Tom E. Wingfield

Academic Editor

PLOS One
